# Chondroitin sulfate shapes the unfolded protein response in osteosarcoma to sustain cancer cell survival during ER stress

**DOI:** 10.1038/s41419-026-09151-9

**Published:** 2026-08-01

**Authors:** Maj Sofie Ørum-Madsen, Jessica Pihl, Thomas M. Clausen, Charlotte Spliid, Anne-Chloé Dhez, Hai-Feng Zhang, Yen-Hsi Chen, Elena Ethel Vidal-Calvo, Tobias Gustavsson, Michael Lizardo, Irina Nelepcu, Joey Lo, Jeffrey D. Esko, Htoo Zarni Oo, Alberto Delaidelli, Mette Ørskov Agerbæk, Anne Steinø, Nader Al Nakouzi, Ali Salanti, Poul Sorensen, Mads Daugaard

**Affiliations:** 1 Vancouver Prostate Centre, Vancouver, BC Canada; 2 Department of Urologic Sciences, University of British Columbia, Vancouver, BC Canada; 3 Department of Immunology and Microbiology, Centre for translational Medicine & Parasitology, University of Copenhagen, Copenhagen, Denmark; 4 Var2 pharmaceuticals Aps, Frederiksberg, Denmark; 5 Department of Cellular and Molecular Medicine, University of California, San Diego, La Jolla, CA USA; 6 John A Burns School of Medicine, University of Hawaii, Honolulu, HI USA; 7 Department of Pathology and Laboratory Medicine, University of British Columbia, Vancouver, BC Canada; 8 Department of Molecular Oncology, British Columbia Cancer Research Institute, Vancouver, BC Canada; 9 Center for Glycomics, Department of Cellular and Molecular Medicine, Faculty of Health Sciences, University of Copenhagen, Copenhagen, Denmark; 10 Department of Pathology, Mass General Brigham, Harvard Medical School, Boston, MA USA

**Keywords:** Glycobiology, Cell death

## Abstract

Tumors must adapt to high levels of endoplasmic reticulum (ER) stress to sustain tumor growth and metastases. The chaperone GRP78 (BiP/HSPA5) is a key component of the unfolded protein response (UPR) and essential for ER stress management and adaptive signaling supporting pro-survival UPR activities. Here, we report that oncofetal chondroitin sulfate (CS) glycosaminoglycans are required for ER stress adaptation in osteosarcoma. When osteosarcoma cells encounter ER stress, they upregulate 4-*O*-sulfated CS at the expense of other glycosaminoglycans leading to a reconfiguration of the glycocalyx in favor of an oncofetal CS subtype. Genetic ablation of the CS synthesis pathway impairs the UPR by preventing osteosarcoma cells from mounting GRP78 expression in response to ER stress. CS deficiency makes osteosarcoma cells hypersensitive to inhibition of GRP78 under both ambient and ER stress conditions, and acute ER stress drives CS-defective osteosarcoma cells into an apoptotic cell death that can be rescued by re-instating CS 4-*O*-sulfation capacity. This has direct implications for the metastatic progression of osteosarcoma, whereby oncofetal CS protects dissociated osteosarcoma cells from *anoikis* to allow pulmonary colonization in mice. Our data identify CS glycosaminoglycans as a critical component of the UPR that permits osteosarcoma cells to manage ER stress.

## Introduction

Most solid tumors share an ‘oncofetal’ chondroitin sulfate (CS) signature with long and distinctly 4-*O*-sulfated CS chains that resemble CS found in the placenta and in the fetal compartment during pregnancy [1]. While the presence of oncofetal CS in the placenta is well-documented, the underlying reasons for its reappearance during cancer progression remains enigmatic [1–5]. Functional assessment of CS in cancer indicates that tumors likely reconfigure the CS glycocalyx to manage intrinsic and extrinsic stress, imposed by high replication and metabolic activity, and a reactive tumor microenvironment [3, 6].

Many stress insults, including replication and metabolic stress in cancer, affect the protein-folding capacity of the endoplasmic reticulum (ER), leading to acute and persistent ER stress conditions that require hasty resolutions for cell survival [7, 8]. To manage and adapt to ER stress, cancer cells elicit the unfolded protein response (UPR) program of which the glucose-regulated protein 78 (GRP78, also known as BiP/HSPA5) is a key component [9, 10]. In osteosarcoma, but also in other solid tumors, GRP78 is required for metastatic disease progression [11–15]. Inhibition of GRP78 extends survival in preclinical metastatic cancer models [11, 12, 15], and high GRP78 expression predicts worse patient outcome for osteosarcoma patients [16], consistent with observations in other cancer types [17–25]. While the role of GRP78 during ER stress is well understood, the mechanisms by which the UPR supports tumor cell viability and fitness are more diverse than previously appreciated.

A correlative relationship between CS, ER stress [26], and oxidative stress [27] has been described post-brain trauma where CS is massively deposited [27–31]. Also, proteoglycan core proteins, particularly neurocan, versican, brevican, and neural/glial antigen 2 are highly up-regulated following central nervous system (CNS) injury [31–35]. Moreover, the hexosamine biosynthetic pathway (HBP) that generates uridine diphosphate N-acetylglucosamine (UDP-GlcNAc) sugars for glycan synthesis has been linked to the UPR in cardiac ischemia reperfusion injury models [36]. It is therefore plausible that oncofetal CS could play a functional role in ER stress management of cancer cells, offering a potential explanation to why most solid tumors present with high oncofetal CS deposits [1, 37]. To examine this idea, we investigated the regulatory and functional relationships between oncofetal CS and ER stress in osteosarcoma. We identify CS glycosaminoglycans as essential for UPR signaling and for osteosarcoma cells to adapt to ER stress.

## Results

### Chondroitin sulfate expression correlates with ER stress in osteosarcoma

We have previously observed that several sarcoma subtypes display high oncofetal CS contents [1]. Building on this observation, we examined the binding of an oncofetal CS-specific reagent, recombinant VAR2CSA (rVAR2) [1], to human primary osteosarcoma specimens (TMA *n* = 80 osteosarcoma tissue cores). High levels of oncofetal CS epitopes (positive score 2–3) were detected in most osteosarcoma tumors (85%), including low grade tumors (Supplementary Fig. S1A, Supplementary Table S1). We next evaluated expression of oncofetal CS on a panel of human osteosarcoma cell lines using a flow cytometry-based assay. We observed a concentration-dependent rVAR2 binding to all cell lines, and this binding was inhibited by competition with purified soluble 4-*O*-sulfated CS chains and by CS ablation in U2-OS cells genetically knocked out for B4GALT7, a key enzyme for CS synthesis (Supplementary Fig. S1B). These data indicate that oncofetal CS epitopes are broadly expressed in osteosarcoma tumors and accessible on the surface of osteosarcoma cancer cells.

**Fig. 1:**
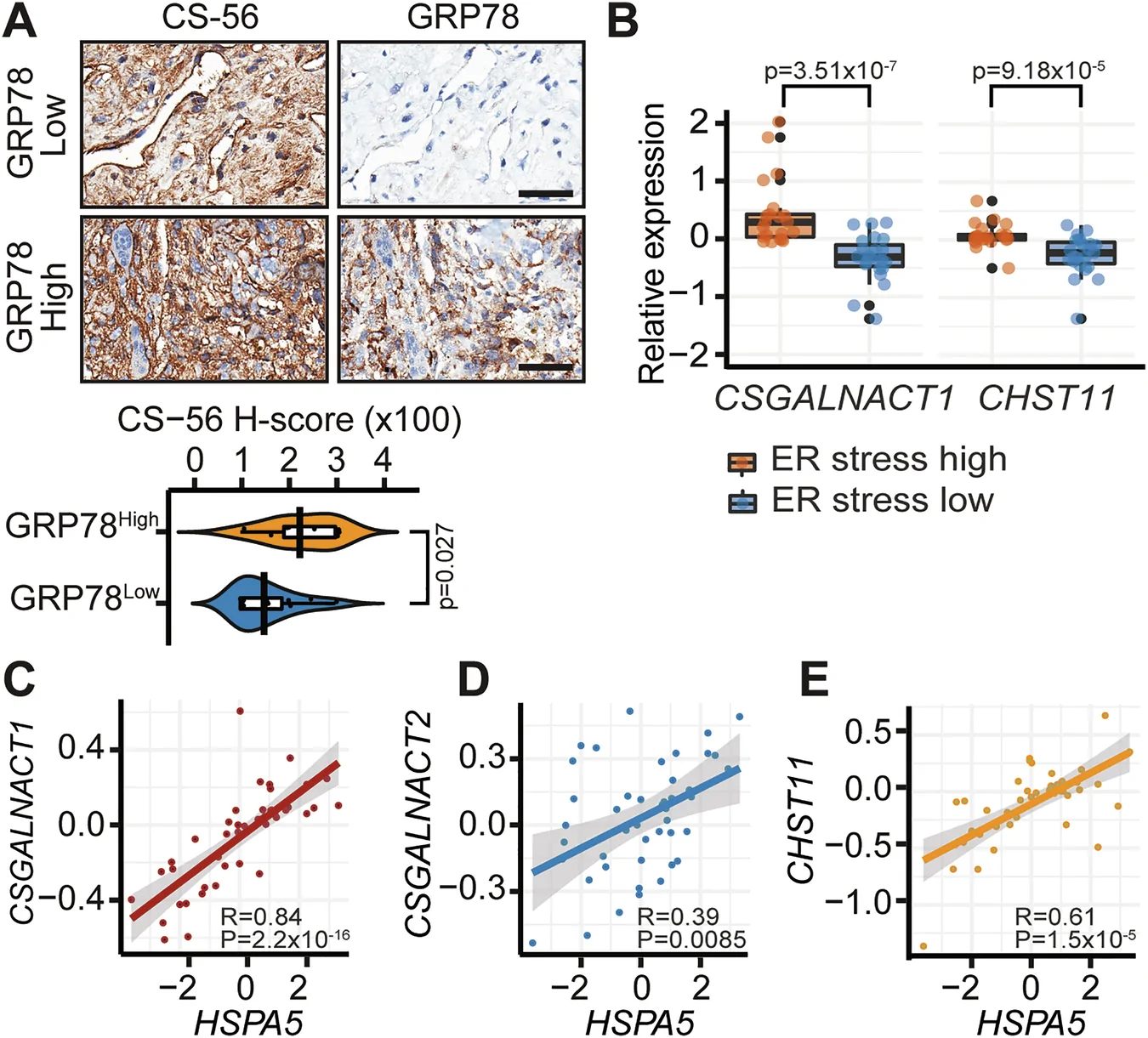
Chondroitin sulfate expression correlates with ER stress in osteosarcoma tumors. **A** Representative IHC images of osteosarcoma tumors stained for sulfated chondroitin sulfate (CS) (CS-56) or GRP78. The scale bar represents 50 μm (upper panel). Violin plot (median ± interquartile range) representing CS-56 H score as a function of high or low GRP78 immunoreactivity in osteosarcoma patient tumors, *n* = 41 (lower panel), *P* = 0.027 (Wilcoxon signed-rank test). **B** Normalized gene expression data in osteosarcoma patient tumors (*n* = 44) were accessed from a previous study GEO (GSE19276). Hierarchical clustering was performed using Euclidean distance and “Ward.D2” clustering method in R. Expression levels of all enzymes involved in chondroitin sulfate synthesis were assessed in two groups based on their endoplasmic reticulum (ER) stress profile (ER stress high vs ER stress low). *CSGALNACT1* and *CHST11* mRNA expression were significantly higher in ER stress high tumors compared to ER stress low tumors. Spearman correlation test *P*-values are indicated on the plots. Scatterplots and correlation analysis accessing the relationship between *HSPA5* (GRP78) vs **C** *CSGALNACT1*, **D** *CSGALANT2*, or **E** *CHST11* were performed on same the dataset as shown in (**B**). *P*-values and Spearman rank coefficients are indicated on the scatterplots.

Given the potential link between CS and ER stress observed in CNS pathologies [26–31], we next analyzed CS expression in the context of key components of the UPR, a well-known cellular pathway activated in response to ER stress. GRP78 was used as the primary ER stress readout because it is the master chaperone of the UPR and a known determinant of osteosarcoma metastatic fitness [15]. We first stained a human osteosarcoma TMA (Supplementary Fig. S2, Supplementary Table S2, *n* = 22) for paired expression of GRP78 (Supplementary Fig. S2A, B) and sulfated CS content (using the CS-56 antibody) (Supplementary Fig. S2C, D). High GRP78-expressing (score 2 and 3) primary osteosarcoma tumors presented with increased accumulation of sulfated CS (*P* = 0.027) (Fig. 1A). These data indicate that a portion of osteosarcomas display ER stress and that this phenomenon is associated with high CS content.

We next examined mRNA expression of various canonical UPR signaling factors in osteosarcoma tumors (*n* = 44, Supplementary Fig. S3) [38] and grouped the tumors into UPR^high^ or UPR^low^ expression signatures based on 11 UPR genes (Supplementary Fig. S3A). Bivariate analyses of glycosaminoglycan biosynthetic enzymes revealed a selective association between the UPR and CS, rather than heparan sulfate (HS), biosynthesis (Supplementary Figs. S3B, S4). Specifically, UPR^high^ tumors exhibited significantly elevated mRNA expression of the 4-*O* sulfotransferase *CHST11* (*P* = 9.18 × 10⁻⁵) and the CS elongation enzyme *CSGALNACT1* (*P* = 3.51 × 10^−7^) (Fig. 1B and Supplementary Fig. S3B), whereas expression of enzymes involved in HS biosynthesis showed no comparable enrichment. These data indicate that ER stress may preferentially promote the synthesis and 4-*O* sulfation of CS chains, rather than HS, suggesting a selective remodeling of the tumor glycocalyx toward 4-*O*-sulfated CS under conditions of elevated UPR activity. Notably, *HSPA5* (the gene encoding GRP78) expression correlated positively with the CS elongation enzymes *CSGALNACT1* (Fig. 1C, (*P* = 2.2 × 10^−16^)), C*SGALNACT2* (Fig. 1D, (*P* = 0.0085)), and the 4-*O* sulfotransferase *CHST11* (Fig. 1E, (*P* = 1.5 × 10⁻⁵)). Combined, these data show that sulfated CS is highly expressed in osteosarcoma and correlate with ER stress signatures.

### ER stress prompts a reconfiguration of glycosaminoglycans in favor of oncofetal chondroitin sulfate

**Fig. 2:**
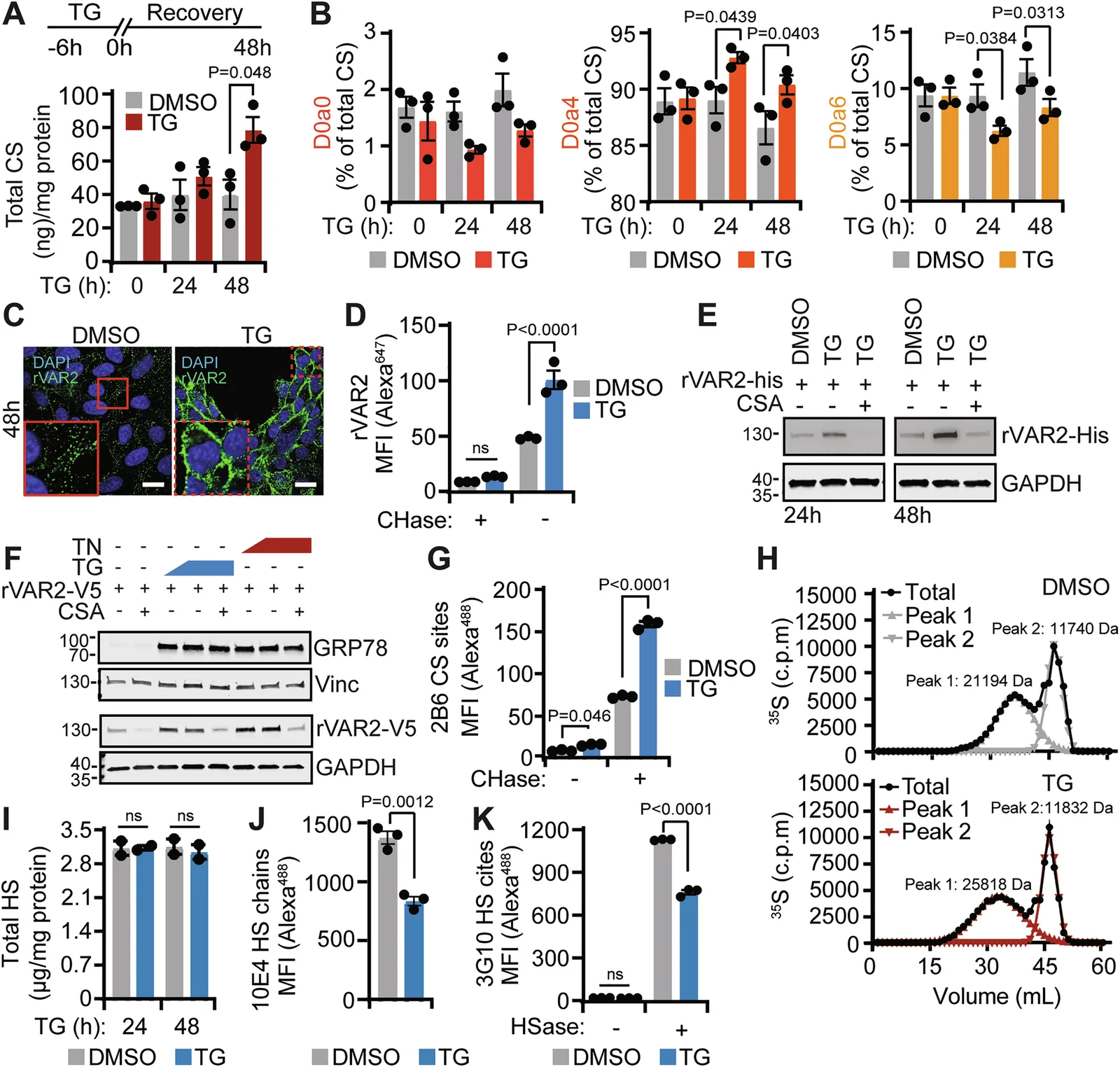
ER stress prompts a reconfiguration of glycosaminoglycans in favor of oncofetal CS subtype. **A**, **B** Glycosaminoglycans (GAG) were digested with chondroitinase ABC (CHase) to generate disaccharides (dp2) that were separated with reverse-phase HPLC (RP-HPLC) and compared to dp2 standards. **A** Absolute quantification of CS dp2 relative to protein content of MNNG cells treated with thapsigargin (TG, 50 nM) or vehicle (DMSO) for 6 h (pulse) with subsequent recovery for 0 h, 24 h, or 48 h (as shown in graphics), the graph represents mean ± SEM, *n* = 3, *P* = 0.0048 1-way ANOVA. **B** Structural analysis of the CS dp2 (D0a0 (left), D0d4 (middle), and D0d6 (right), from (**A**). Data is presented as relative abundance (% dp2 to total CS); graph represents mean ± SEM, *n* = 3, *P*-values were calculated for each dp1 at each timepoint using Student’s *t* test. **C** Representative confocal images of V5-tagged recombinant VAR2 (rVAR2-V5) binding to MNNG cells recovering (48 h) from TG/DMSO pulse, detected by anti-V5 and anti-rabbit-488 (green) antibodies, nuclei visualized with DAPI (blue), *n* = 3. Scale bars represent 20 μm. **D** Binding of Alexa647 labeled rVAR2 to MNNG cells recovering (48 h) from TG/DMSO pulse including ± pre-digestion with CHase as detected by flow cytometry, graph represents mean MFI ± SEM, *n* = 3, *P* < 0.0001 (2-way ANOVA). **E** Binding of rVAR2-his to MNNG cells, recovering (48 h) from TG/DMSO ± chondroitin sulfate A (CSA) competition as detected by Western Blotting, representative blots, *n* = 3. GAPDH serves as protein loading control. **F** Binding of rVAR2-V5 to MG-63 cells exposed to (24 h) TG (0.5 µM or 1 µM), TN (2 µg/mL or 4 µg/mL) or vehicle (DMSO) ± CSA competition as detected by Western Blotting, representative blots, *n* = 3. **G** Detection of CS neoepitopes by 2B6 binding in MNNG cells recovering (48 h) from TG/DMSO pulse ± pre-digestion with CHase as detected by Alexa 647 using flow cytometry, graph represents mean MFI ± SEM, *n* = 3, *P*-values obtained by 2-way ANOVA. **H** Chain length analysis of 35S-labeled CS by size exclusion of MNNG cells recovering (48 h) from a TG/DMSO pulse, representative image of *n* = 3. **I** GAGs were digested with heparinase to generate dp2 that were separated with RP-HPLC and compared to dp2 standards. Graph indicates absolute levels of heparan sulfate (HS relative to protein content) in MNNG cells recovering (24 h or 48 h) from TG/DMSO pulse, graph represents mean ± SEM, *n* = 2. Ns *P*-value obtained by Student’s *t* test. **J** HS chains detected by 10E4 binding to MNNG cells recovering (48 h) from a pulse of TG/DMSO as detected by Alexa 488 using flow cytometry, graph represents mean MFI ± SEM, *n* = 3, *P* = 0.0012 (Student’s *t* test). **K** HS neoepitopes detected by 3G10 binding to ± heparinase III (HSase) pre-digested MNNG cells recovering (48 h) from a pulse of TG/DMSO as detected by Alexa 488 using flow cytometry, graph represents mean MFI ± SEM, *n* = 3, *P* < 0.0001 (2-way ANOVA).

To investigate a potential causative relationship between ER stress and CS synthesis in osteosarcoma, we pharmacologically induced sub-lethal ER stress in MNNG osteosarcoma cells using thapsigargin (50 nM, TG) for 6 h and subsequently evaluated the absolute CS content and its specific disaccharide composition using HPLC at 24 h or 48 h. GRP78 expression, indicative of UPR signaling, was verified using western blotting (Supplementary Fig. S5A, B). The cellular CS content significantly increased in response to ER stress compared to vehicle (DMSO)-treated controls in a time dependent manner (Fig. 2A) (*P* = 0.0048). This was confirmed by increased cell surface-binding of the CS-56 antibody to ER-stressed MNNG cells, assessed by flow cytometry (Supplementary Fig. S5C) (*P* < 0.0001). When analyzing the sub fractions of CS, non-sulfated CS disaccharides (D0a0) remained low, while 4-*O*-sulfated (D0a4) CS was increased at 24 h and 48 h (*P* = 0.0439 and *P* = 0.0403, respectively), and 6-*O*-sulfated (D0a6) CS was reduced at 24 h (*P* = 0.0384) and 48 h (*P* = 0.0313) (Fig. 2B). The selective increase in 4-*O*-sulfation indicated that oncofetal CS epitopes might be enriched after ER stress.

To confirm this, rVAR2 lectin binding to MNNG cells was assessed. Confocal microscopy of non-permeabilized cells showed increased cell surface presentation of oncofetal CS under conditions of ER-stress (Fig. 2C). Similar results were obtained by flow cytometry where pre-digestion of CS with chondroitinase ABC (CHase) removed rVAR2 binding as a control for oncofetal CS specificity (Fig. 2D) (*P* < 0.0001). Additionally, immunoblotting for rVAR2-his confirmed an increased binding of rVAR2 to oncofetal CS after ER stress in MNNG cells, which was reduced by competition with soluble 4-*O*-sulfated CS chains (Fig. 2E). These data indicate that the cellular glycocalyx becomes enriched for oncofetal CS in response to ER stress. Tunicamycin (TN), a mechanistically different type of ER stress inducer, and heat shock, also elevated cellular oncofetal CS levels in MNNG cells as visualized by confocal microscopy (Supplementary Fig. S5D, E). Similar results were obtained in MG-63 cells cultured in sublethal doses of TG and TN for 24 h prior to rVAR2 probing and immunoblotting. Here, cell surface rVAR2 binding was increased in MG-63 cells upon ER stress (confirmed by GRP78 protein expression) as compared to vehicle controls, and free 4-*O*-sulfated CS chains competitively inhibited rVAR2 binding (Fig. 2F). To determine whether the observed phenotype resulted from an increase in the amount and/or length of CS chains, we performed size-exclusion chromatography on radiolabeled cellular CS and assessed cell surface binding of the 2B6 antibody. The 2B6 antibody specifically recognizes 4-*O*-sulfated unsaturated disaccharide neoepitopes that are exposed at the non-reducing ends of CS chains following CHase digestion [39, 40]. Indeed, elevated cell surface 2B6 reactivity was observed after ER stress, indicating that ER stress triggers an increase in the number of CS chains on cell surface proteoglycans (*P* < 0.0001) (Fig. 2G). In addition, we detected a shift towards longer CS populations in response to ER stress (Fig. 2H). These data indicate that ER stress induces CS biosynthesis, resulting in both an increased number of CS chains and in extended CS chain length. We next assessed HS levels to determine whether ER stress broadly affected sulfated GAGs or whether the response was selective for CS, as suggested by the gene expression patterns observed in UPR^high^ tumors (Supplementary Fig. S3A, B). Notably, no changes in total cellular HS levels upon ER stress could be observed by HPLC (Fig. 2I). On the contrary, flow cytometric analysis of HS surface levels assessed by 10E4 antibody (*P* = 0.0012) binding (Fig. 2J), and HS attachment sites assessed by 3G10 binding (*P* < 0.0001) (Fig. 2K), revealed decreased HS levels under ER stress compared to controls. As such, MNNG cells down-regulate cell surface HS levels after ER stress with a decrease in the number of HS modified sites. Combined, these data show that ER stress leads to a selective increase in oncofetal 4-*O*-sulfated CS in the osteosarcoma glycocalyx.

### Chondroitin sulfate enables GRP78 expression during ER stress

**Fig. 3:**
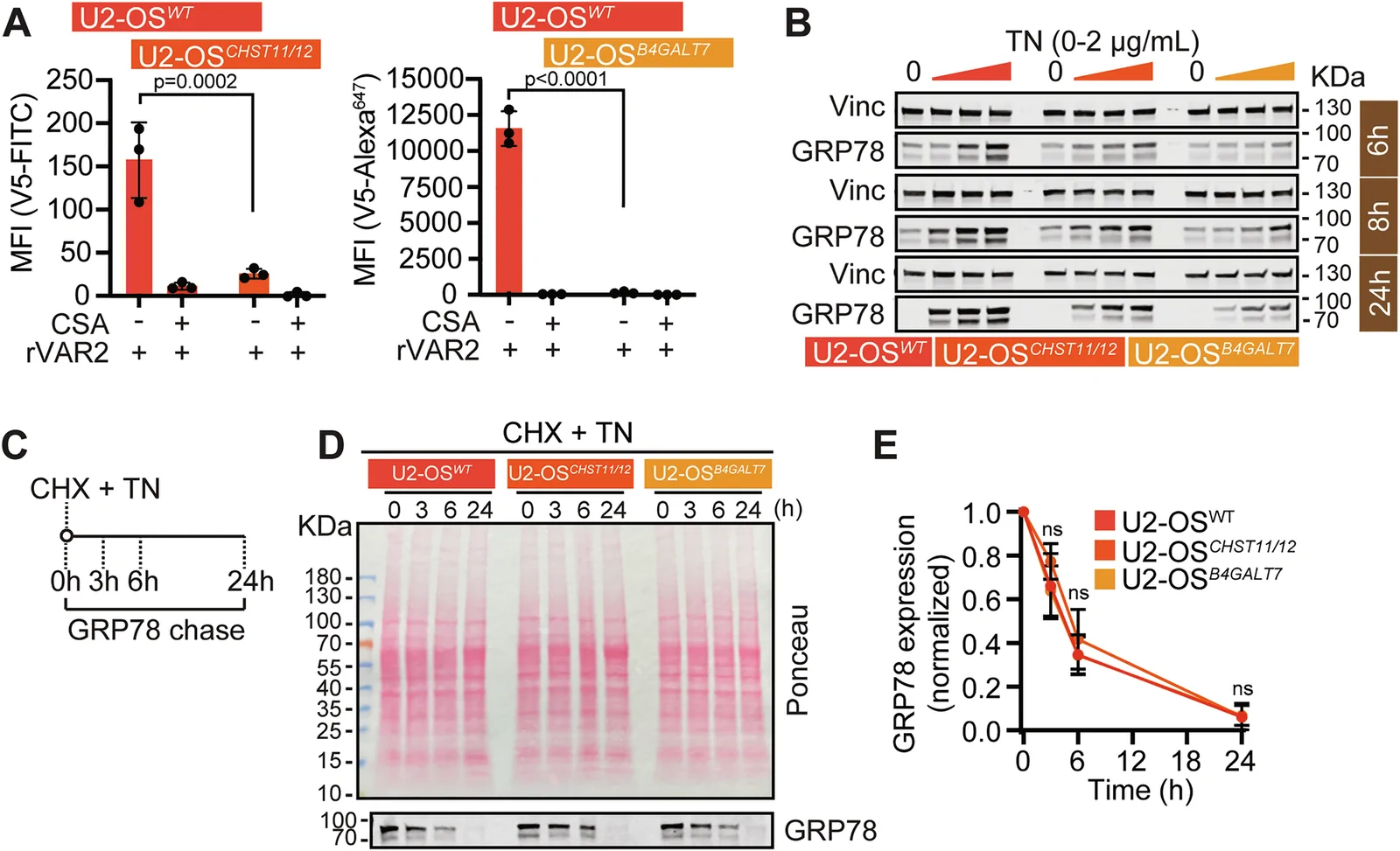
Chondroitin sulfate enables GRP78 expression during ER stress. **A** rVAR2-V5 binding (100 nM) ± CSA competition to U2-OS^*CHST11/12*^ (left) and U2-OS^*B4GALT7*^ (right) cells compared to U2-OS^WT^ as assessed by flow cytometry using anti-V5-FITC (left) or anti-V5-Alexa647 (right) for detection, *P* < 0.0001 (2-way ANOVA). **B** Western blot analysis of GRP78 expression in U2-OS isogenic cells upon dose escalations of tunicamycin (0.2 µg/mL, 1 µg/mL, or 2 µg/mL TN) and vehicle controls (DMSO) at 6 h, 8 h, or 24 h. Vinculin (vinc) was used as protein loading control, blots representative of *n* = 3. **C** Graphic illustration of the cycloheximide (CHX, 40 µg/mL) chase assay applied to evaluate GRP78 half-life during TN (1 µg/mL) treatment for up to 24 h. Cell lysate harvesting timepoints are indicated. **D** Representative blot showing (upper) Ponceau (total protein) and (lower) Western blot analysis of GRP78 expression in U2-OS isogenic cells over time. **E** Densitometric quantification of GRP78 protein levels over time, normalized to total protein and represented relative to time 0. Data shown are mean ± SD of *n* = 3 (non-significant, 1-way ANOVA).

We next investigated the function of oncofetal CS in the ER stress response. We first generated an isogeneic model system that would allow us to assess the importance of oncofetal CS in osteosarcoma upon ER stress. U2-OS cells were knocked out for the 4-*O*-sulfotransferases CHST11 and CHST12 (U2-OS^*CHST11/12*^), or the beta-1,4-galactosyltransferase B4GALT7 (U2-OS^*B4GALT7*^) (Supplementary Fig. S6A). Compared to U2-OS^WT^ cells, U2-OS^*CHST11/12*^ and U2-OS^*B4GALT7*^ cells displayed low 4-*O*-sulfated CS levels (Supplementary Fig. S6B). Consequently, while U2-OS^WT^ cells interacted with the oncofetal CS binding-reagent, rVAR2, U2-OS^*CHST11/12*^ and U2-OS^*B4GALT7*^ cells displayed only minimal reactivity to rVAR2 (Fig. 3A). These data confirm that the isogeneic U2-OS cells KO for CHST11/12 or B4GALT7 have reduced oncofetal CS presentation. We next investigated how these isogeneic cells responded to ER stress. U2-OS^WT^, U2-OS^*CHST11/12*^ and U2-OS^*B4GALT7*^ cells were subjected to TN and examined for their ability to activate an ER stress response. While U2-OS^WT^ cells rapidly upregulated GRP78 expression in response to increasing concentrations of TN, U2-OS^*CHST11/12*^ and U2-OS^*B4GALT7*^ cells were unable to mount a durable GRP78 response (Fig. 3B and Supplementary Fig. S6C). Similar results were observed for TG (Supplementary Fig. S7). Given that GRP78 can be secreted or re-localized to the cell surface in cancer [41–43], we next examined whether altered trafficking through secretion could account for the observed differences in cellular GRP78 levels. Because CS is synthesized in the Golgi, and CD44 variant isoforms - known CS proteoglycans - have been implicated in GRP78 cell-surface localization [44, 45], we investigated whether loss of CS affected GRP78 trafficking or secretion. Soluble GRP78 (sGRP78) was not elevated in conditioned media from CS-deficient cells under stress (Supplementary Fig. S8A, B). We also assessed transcriptional regulation of GRP78 in U2-OS^WT^, U2-OS^*CHST11/12*^, and U2-OS^*B4GALT7*^ cells after ER stress. We observed no appreciable differences in GRP78 (*HSPA5*) mRNA levels between the isogenic cell lines during ER stress conditions (Supplementary Fig. S9A, B). We next investigated if CS might affect GRP78 protein translation during ER stress. Inhibition of protein translation by cycloheximide during ER stress reduced GRP78 in U2-OS^WT^ cells to levels similar to U2-OS^*CHST11/12*^ and U2-OS^*B4GALT7*^ cells (Fig. 3C–E and Supplementary Fig. S10A–D). Combined, these results suggest that oncofetal CS supports the defense against ER stress in osteosarcoma by enabling stress-induced GRP78 protein expression.

### Chondroitin sulfate deficiency sensitizes cells to GRP78 depletion

**Fig. 4:**
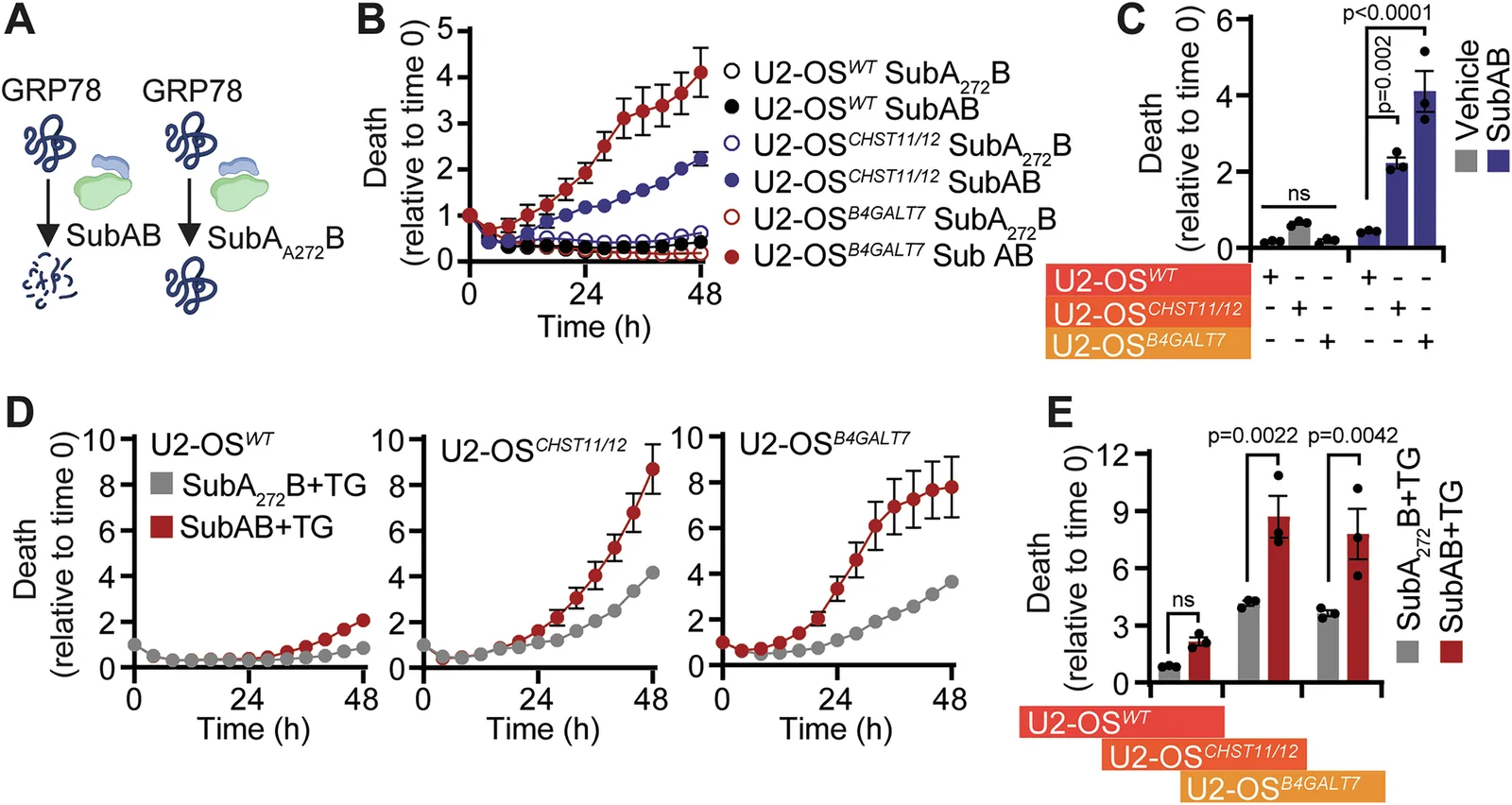
Chondroitin sulfate deficiency sensitizes cells to GRP78 depletion. **A** Graphical illustration of the Subtilase AB (subAB) mechanism of action with specific degradation of GRP78 compared to the inert SubA_A272_B variant, created with Biorender. **B** Normalized death levels (relative to time 0) measured in real-time in U2-OS isogenic cell lines cultured in the presence of subAB or the inert subA_A272_B (100 ng/mL) using the IncuCyte. Data shown are mean of 3 technical replicates representative of *n* = 3. **C** Death (relative to time 0) quantified at 48 h from (**D**), mean of 3 technical replicates representative of *n* = 3, *P* = 0.002 and *P* < 0.0001 (2-way ANOVA). **D** Death levels (relative to time 0) measured in real-time in U2-OS isogenic cell lines cultured in the presence of subAB or subA_A272_B (100 ng/mL) ± thapsigargin (TG, 50 nM) using the IncuCyte. Data shown are mean of 3 technical replicates representative of *n* = 3. **E** Death (red area relative to time 0) quantified at 48 h from (**F**), mean of 3 technical replicates representative of *n* = 3, *P* = 0.0022 and *P* = 0.0042 (2-way ANOVA).

We next investigated the functional importance of GRP78 and CS during ER stress in osteosarcoma. We assessed cellular fitness (i.e. death measured by propidium iodide (PI) uptake) in response to subtilase AB5 (subAB5), a site-directed serine-like protease that specifically degrades GRP78 [46], with or without ER stress (TG and TN treatment) using an inactive SubAA272B enzyme as a control (Fig. 4A). Under ambient conditions, U2-OS^*CHST11/12*^ (*P* = 0.002) and U2-OS^*B4GALT7*^ (*P* < 0.0001) cells were more sensitive to GRP78 degradation as compared to U2-OS^*WT*^ cells (Fig. 4B, C). These data indicate that cells lacking an intact CS glycocalyx have a higher basic dependency on GRP78 even though they have similar basal GRP78 expression levels under ambient conditions. When subjected to ER stress, U2-OS^*CHST11/12*^ (*P* = 0.0022) and U2-OS^*B4GALT7*^ (*P* = 0.0042) cells were even more sensitive to GRP78 degradation as compared to U2-OS^*WT*^ cells (Fig. 4D, E). Combined, these data suggest that impaired CS expression increases cellular dependence on GRP78, particularly under ER stress conditions.

### CS deficiency sensitizes tumor cells to ER stress-induced apoptosis

**Fig. 5:**
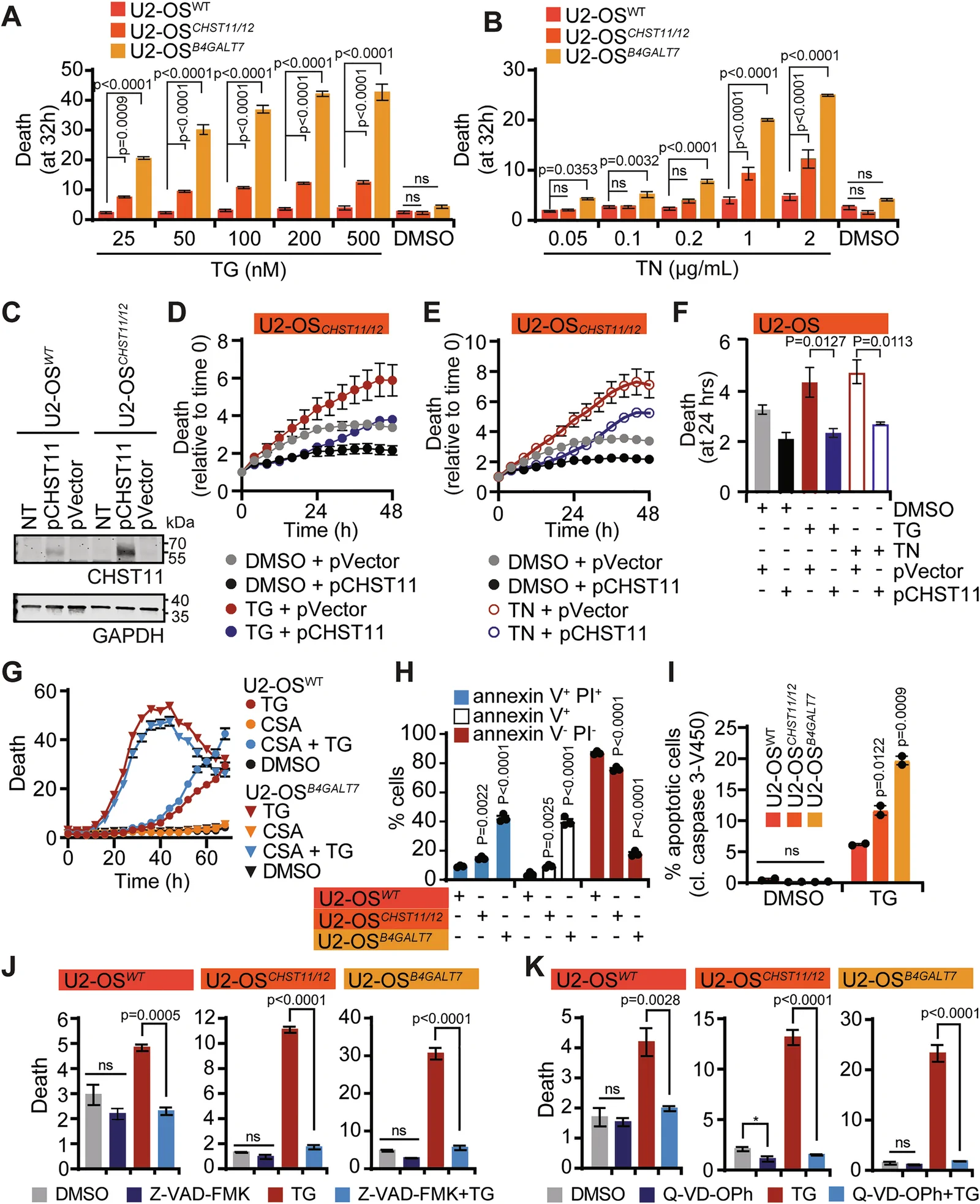
CS deficiency sensitizes osteosarcoma cells to ER stress-induced cell death. Death as measured by PI uptake using IncuCyte (red area (µm^2^ × 10^3^/well) in U2-OS isogenic cell lines treated with **A** thapsigargin (TG, 25–500 nM), **B** (TN, 0.05–2 µg/mL) or vehicle (DMSO) at 32 h. Data shown are mean of 3 technical replicates ±SEM, representative of *n* = 3–6, *P*-values obtained by 2-way ANOVA. **C** Western blot analysis of CHST11 expression in U2-OS^WT^ and U2-OS^*CHST11/12*^ cells transfected (72 h) with CHST11 plasmid (pCHST11) or empty control plasmid (pVector). GAPDH was used as protein loading control. **D**–**F** Normalized death (relative to time 0) measured real-time in U2-OS^*CHST11/12−/−*^ cells transfected with pVector or pCHST11 and treated with **D** TG (200 nM) or **E** TN (1 µg/mL) or vehicle (DSMO) using IncuCyte. **F** Death levels in U2-OS^*CHST11/12*^ at 24 h post TG/TN treatment. *P*-values obtained by 1-way ANOVA. **G** U2-OS^WT^ and U2-OS^*B4GALT7*^ cells were ± pre-treated for 24 h with CSA (100 µg/mL) prior to culturing with TG (500 nM) or vehicle (DMSO) in the continues presence of CSA. Death was assessed in real-time using the IncuCyte system. **H** Flow cytometric apoptosis detection in U2-OS isogenic cells treated with TG (200 nM) or vehicle (DMSO) for 32 h using propidium iodide and Annexin V-FITC co-staining, data shown are mean percentage of negative/single/double positive cell populations of 3 technical replicates representative of *n* = 3, *P*-values were obtained by 2-way ANOVA. **I** Flow cytometric analysis of cleaved caspase 3 expression as detected by active-caspase3-V450 after culturing U2-OS isogenic cell lines for 32 h in the presence of TG (200 nM) or vehicle (DMSO), data shown are mean ± SEM of 2 technical replicates representative of *n* = 3, *P*-values were obtained by 1-way ANOVA. **J**–**K** Death levels in U2-OS isogenic cell lines cultured in the presence of pan-caspase inhibitors (**J**) Z-VAD-FMK (10 µM) ± TG (200 nM) or (**K**) Q-VD-OPh (20 µM) ± TG (200 nM) or vehicle (DMSO) at 32 h, PI uptake was measured using the IncuCyte system. Graphs show mean red area ± SEM of 3 technical replicates, representative of *n* = 3, *P*-values were obtained by 1-way ANOVAs.

To further assess the function of oncofetal CS during ER stress, live cell imaging analyses were applied to track cell death (PI uptake) in the U2-OS isogenic cells over time when exposed to incremental concentrations of pharmacological ER stress inducers. Parental U2-OS^WT^ cells with an intact GAG repertoire were more resistant to TG (Fig. 5A and Supplementary Fig. S11A) and TN (Fig. 5B and Supplementary Fig. S11B) as compared to both U2-OS^*CHST11/12*^ and U2-OS^*B4GALT7*^ cells. As expected, cells with complete KO of CS (U2-OS^*B4GALT7*^) were even more sensitive to ER stress than cells with decreased 4-*O*-sulfation (U2-OS^*CHST11/12*^). To confirm the specific impact of 4-*O*-sulfation in protecting tumor cells against ER stress, we restored CHST11 expression in U2-OS^*CHST11/12*^ cells (Fig. 5C). CHST11 reconstitution in U2-OS^*CHST11/12*^ cells rescued the cell death induced by both TG (*P* = 0.0127) and TN (*P* = 0.0113) (Fig. 5D–F). Ectopic overexpression of CHST11 in MG-63 and MNNG cells produced a similar phenotype, characterized by reduced cell death relative to empty vector controls, consistent with a gain-of-function effect of CHST11 (Supplementary Fig. S12). Pre-incubation with soluble 4-*O*-sulfated CS chains (100 µg/mL CSA, average molecular weight 20 kDa) was unable to rescue cell death of U2-OS^*CHST11/12*^ and U2-OS^*B4GALT7*^ cells induced by ER stress, suggesting that 4-*O*-sulfated CS needs to be proteoglycan and cell associated to exert its protective effects (Fig. 5G).

To investigate the mechanism of cell death associated with CS loss in osteosarcoma cells under prolonged ER stress, we first used PI/Annexin V flow cytometry to distinguish viable, apoptotic, and necrotic cell populations based on differences in plasma membrane integrity and permeability [47]. U2-OS^*CHST11/12*^ and U2-OS^*B4GALT7*^ cells had higher levels of PI/Annexin V positive cells when treated with TG as compared to parental U2-OS^wt^ cells (Fig. 5H). Also, increased caspase 3 cleavage was observed in both U2-OS^*CHST11/12*^ (*P* = 0.0122) and U2-OS^*B4GALT7*^ (*P* = 0.0009) cells as compared to U2-OS^wt^ cells (Fig. 5I). These data suggest that loss of CS sensitizes U2-OS cells to apoptosis. Indeed, two pan-caspase inhibitors (Z-VAD-FMK and Q-VD-OPH) could completely rescue the death in all three isogenic cell lines caused by prolonged TG treatment as evident by real-time cell imaging (Fig. 5J, K). Similar results were obtained with TN treatment (Supplementary Fig. S13C, D). Importantly, the necroptosis inhibitor NST-1 (Supplementary Fig. S14A, B) and ferroptosis inhibitor FST1 (Supplementary Fig. S14C, D) had no effect on cell survival during ER stress in any of the U2-OS isogenic cell lines. These data suggest that CS protects tumor cells from ER stress-induced apoptosis.

### Chondroitin sulfate determines the metastatic potential of osteosarcoma cells

**Fig. 6:**
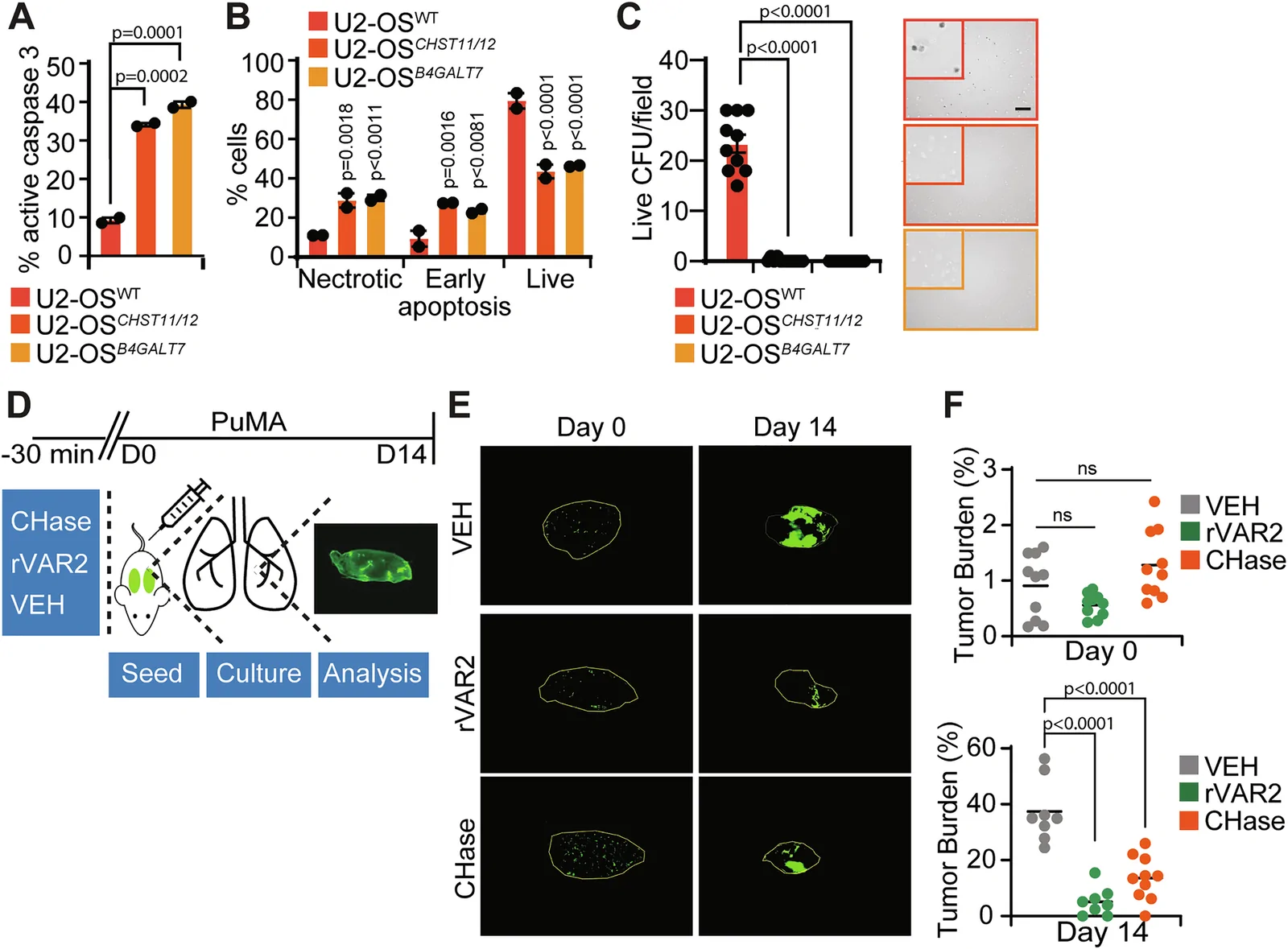
Chondroitin sulfate determines the metastatic potential of osteosarcoma cells. Anoikis assessment through **A** Cleaved caspase 3-V450 detection and **B** PI and Annexin V co-staining of U2-OS isogenic cell lines cells cultured for 15 h in low attachment plates assessed by flow cytometry. Data is mean of 2 technical replicates ±SEM, representative of *n* = 3. *P*-values were obtained using 1 and 2-way ANOVAs. **C** Soft agar colony formation of isogenic U2-OS cell lines. Live colonies detected by MTT assay. Data are means of live colony forming units (CFU) of 8–10 images taking from 3 technical replicates ±SEM representative, representative of *n* = 3, *P* < 0.0001 (1-way ANOVA). Scalebar represents 500 µm. **D** Experimental workflow of the Ex vivo pulmonary metastasis assay with *e*GFP expressing MNNG cells (*e*GFP^+^) in murine lung tissue. Cells were either pre-digested with chondroitinase ABC (Chase) or pre-incubated with recombinant VAR2 (rVAR2) prior to inoculation in the tail vein. PBS (vehicle) treated *e*GFP^+^ MNNG acted as controls. Lung sections were subsequently harvested and grown ex vivo. **E** Fluorescence of *e*GFP^+^ MNNG (green) in lung tissue at day 0 (D0) and day 14 (D14), images taken with 5X magnification. **F** (upper) Tumor burden (Mean fluorescent values relative to total lung slice area after injection of the cells at D0 of the respective groups, 7–10 lung pieces/group with min/max values representative of 2–3 independent experiments. (Lower) Tumor burden at D14. *P* < 0.0001 (1-way ANOVA).

Persistent ER stress is intimately linked to metastases formation [48, 49]. In light of the apoptotic protection mediated by 4-*O*-sulfated CS demonstrated in this study, we hypothesized that 4-*O*-sulfated CS may similarly promote resistance to *anoikis*, a caspase-dependent apoptotic process induced by detachment from the extracellular matrix or cell–cell contacts during metastasis. The U2-OS isogenic cell lines were cultured in low attachment plates and assessed for caspase 3 cleavage in flow cytometry (Fig. 6A). Compared to U2-OS^wt^ with an intact GAG repertoire, both U2-OS^*CHST11/12*^ and U2-OS^*B4GALT7*^ cells displayed significantly higher levels of caspase 3 cleavage (U2-OS^*CHST11/12*^ *P* = 0.0002; U2-OS^*B4GALT7*^ *P* = 0.0001) when grown in suspension. This observation was supported by PI/Annexin V assay, which revealed significantly elevated levels of apoptosis/necrosis in both U2-OS^*CHST11/12*^ (*P* = 0.0018, *P* = 0.0016, and *P* < 0.0001) and U2-OS^*B4GALT7*^ (*P* = 0.0011, *P* = 0.0081, and *P* < 0.0001) cultures compared to parental U2-OS^wt^ cells (Fig. 6B).

Unlike U2-OS^WT^, U2-OS^*CHST11/12*^ (*P* < 0.0001) and U2-OS^*B4GALT7*^ (*P* < 0.0001) cells were incapable of forming live colonies in soft agar, underscoring the role of 4-*O*-sulfated CS in supporting anchorage independent cell growth in osteosarcoma (Fig. 6C). Given that the cytoprotective effects of CS during stress have previously been attributed to its antioxidant properties [50], and that oxidative stress regulation is closely linked to *anoikis* resistance [48, 49, 51], we investigated whether the antioxidants Trolox and NAC could prevent *anoikis* induction following CS loss. However, neither Trolox nor NAC could rescue this phenotype in CS knock-out cells indicating mechanisms beyond oxidative stress (Supplementary Fig. S15A, B). Finally, to directly investigate the importance of oncofetal CS in ER challenged metastatic lung colonization over time, we used an ER stress proficient in vivo to ex vivo lung explant mouse model referred to as the pulmonary metastasis assay (PuMA) [15, 52]. In this model, tail vein injected *e*GFP-labeled tumor cells are allowed to seed in the lungs of mice, whereafter metastatic clones are tracked live in resected lungs grown ex vivo for 14 days. This model recapitulates ER stress in a physiologic relevant context [15] and displays GRP78 expression in the ofCS positive metastatic foci (Supplementary Fig. S15C). Indeed, *eGFP* expressing MNNG cells injected into the tail vein of nude mice led to extensive metastatic growth in the lungs (Fig. 6D–F, and Supplementary Fig. S15D). When oncofetal CS chains were masked by saturating concentrations of the rVAR2 lectin prior to tail vein injection, the formation of metastatic foci in the lungs was almost eliminated, while pre-inoculation fitness was unaffected (*P* < 0.0001) (Fig. 6E, F, Supplementary Figs. S15E, F, S16A–D, S17A–D). Similarly, when MNNG cells were pre-treated with CHase enzyme, cleaving the CS chains, prior to injection, metastatic growth was also reduced (*P* < 0.0001) (Fig. 6E, F, Supplementary Figs. S15D, S16A–D). Notably, lung seeding (i.e. *eGFP* levels at time 0) was not affected by rVAR2 or CHase (Fig. 6F, Supplementary Fig. S16C, S17C), suggesting oncofetal CS may be critical for osteosarcoma cell outgrowth in lungs, as opposed to initial seeding. Collectively, these results suggest that CS is required for osteosarcoma *anoikis* resistance and metastatic foci growth in the lungs. Therapeutic targeting of 4-*O*-sulfated CS could therefore offer an exciting intervention point to potentially counter metastatic dissemination of osteosarcoma cells.

## Discussion

Despite aggressive multi-agent chemotherapy regimens, the 5-year survival rate of osteosarcoma patients with metastatic disease remains dismal, with no substantial improvement for decades [53, 54]. The field has reached a therapeutic ceiling with conventional cytotoxic agents, highlighting the urgent need to identify and validate new tumor vulnerabilities that contribute to disease progression. Among these, CS GAGs have emerged as promising therapeutic candidates, and elucidating the regulatory mechanisms that govern their expression may unlock new opportunities for therapeutic intervention.

In this study, we demonstrate that osteosarcomas from children and adolescents abundantly display sulfated CS GAGs, including oncofetal CS moieties that are potentially targetable. These observations extend prior work identifying oncofetal CS as a conserved, pan-cancer target [1, 6, 37, 55, 56]. While numerous studies have linked aberrant glycosylation to ER stress [57–60], the reciprocal relationship observed between the ER stress response and CS GAGs remains largely unexplored. We uncovered a dependence of 4-*O*-sulfated CS for GRP78 expression after ER stress in experimental models. This identifies CS as a previously unrecognized component of the UPR in osteosarcoma where CS biosynthesis is prioritized during ER stress.

ER stress induced both quantitative and qualitative modifications to the cellular GAG architecture resulting in elongation of 4-*O*-sulfated CS chains consistent with increased oncofetal CS expression [4], accompanied by a reduction in HS. The observed changes in cell surface CS levels are particularly striking given that, under normal conditions, the overall abundance of CS in these cells is many folds lower than that of HS. These results are concordant with observations in neurological models, where ER stress [26] and oxidative stress [27, 61] similarly drive CS accumulation, leading to the formation of dense CS-rich extracellular matrices in traumatized CNS tissue [27–31]. Cell surface HS loss has also been shown to accompany UPR activation in pancreatic intra-islet/beta cells [62], paralleling our observation in cancer. Mechanistically, prior studies in non-cancerous models have shown that small-molecule-mediated activation of the UPR transcription factors XBP1 and ATF6 [63] promote transcriptional activation of *CSGALNACT1* and UDP-glucose 6-dehydrogenase (*UGDH)*, respectively, enzymes that promote CS chain initiation and GAG precursor availability [64]. Moreover, UPR signaling and other stress pathways alter Golgi organization and trafficking [65, 66], leading to spatial redistribution of glycosyltransferases into distinct functional microdomains [67, 68] that influence GAG chain elongation, sulfation patterns, and trafficking [69]. Collectively, these data support a model in which ER stress promotes CS biosynthesis through a combined effects on precursor supply, enzyme accessibility, and stress-induced Golgi remodeling, thereby favoring CS production under stress conditions.

Importantly, we identify CS as a regulator of sustained GRP78 protein expression in osteosarcoma cells, specifically in a stress context. CS-deficient cells exhibited markedly reduced GRP78 levels and GRP78 depletion disproportionately impaired the viability of CS-deficient cells, indicating an elevated dependence on GRP78 when the CS-glycocalyx is compromised. GRP78 induction during ER stress depends not only on transcription but also on selective translation when global protein synthesis is suppressed [70]. Translational blockage indeed rescued the GRP78 half-life in CS deficient cells, suggesting that CS contributes to stress adaptive translational control of GRP78. CS-dependent regulation of signaling pathways [71–73], as well as growth factor and receptor dynamics mediated by CS-rich proteoglycans [74, 75] may influence translational efficiency and UPR output. Although the precise mechanism by which CS regulates GRP78 translation remains to be defined, our data indicate that CS is not simply a structural component but actively participates in fine-tuning cellular stress adaptation.

The interdependence of CS, GRP78, and the ER stress response suggests a compensatory survival network that couples intracellular proteostasis with glycocalyx integrity, offering a potential synthetic lethal strategy for therapeutic intervention. Given GRP78’s known roles in osteosarcoma progression [15, 16] and chemoresistance [76, 77], therapeutic targeting of CS may expose a critical weakness in aggressive disease.

Beyond their established roles in tumor progression, including regulation of proliferation, angiogenesis, migration, invasion, and metastasis [73, 78–82], our data indicate that tumor-associated CSPGs are integral components of the cellular stress-adaptation program that enables metastatic competence. Notably, although ER stress increased CS abundance in our system, directly testing the causal contribution of elevated CS to tumor progression remains challenging. CS biosynthesis is governed by a highly coordinated network of Golgi-resident enzymes, and simple overexpression of individual biosynthetic enzymes has been reported to perturb rather than predictably increase GAG production [83]. To circumvent these limitations, we instead employed epitope masking of oncofetal CS using rVAR2, allowing functional interrogation of this glycan structure without perturbing the underlying biosynthetic machinery. Although the involvement of oncofetal CS in metastatic behavior has been appreciated [73], the precise stage(s) of the metastatic cascade governed by CS have remained unresolved. Using the PuMA model to monitor metastatic progression in real time, we demonstrate that blockade of oncofetal CS does not affect initial lung seeding but profoundly impairs subsequent metastatic outgrowth. Notably, GRP78 has previously been identified as a critical dependency for osteosarcoma metastatic outgrowth in the PuMA model [15], underscoring the importance of ER stress adaptation during lung colonization. In this context, our observation that metastatic foci are consistently GRP78-positive and enriched for oncofetal CS, provides a biological link between CS remodeling, ER stress adaptation, and metastatic fitness. These findings position oncofetal CS as a critical determinant of post-intravasation survival and colonization, most likely by conferring resistance to *anoikis* - a key stress burden encountered by disseminating tumor cells.

In conclusion, our work establishes oncofetal CS as a central regulator of osteosarcoma cell survival across multiple dimensions of stress, including ER stress and the stress imposed by the hostile microenvironment encountered during metastatic dissemination. Unlike most protein biomarkers, oncofetal CS arises from stress-induced glycan remodeling that is tightly coupled to tumor adaptability and survival. The strong functional dependence on CS-mediated stress management highlights osteosarcoma as a compelling candidate for emerging oncofetal CS-targeted therapies currently in development [37].

## Materials and methods

### Reagents

Pharmacological ER stressors thapsigargin (Enzo Biochem, #BML-PE180) and tunicamycin (TN) (Sigma Aldrich #7765) were dissolved in dimethyl sulfoxide (DMSO) (Sigma Aldrich, #2438). For cell death mechanism studies, necrostatin-1 (R&D systems, #2324), Z-VAD-FMK (R&D Systems, #FMK001), Q-VD-OPh (R&D Systems, #OPH00101), and ferrostatin-1 (Cayman Chemical Company #17729) were used at indicated concentrations. For antioxidant rescue studies, we used Trolox (Sigma-Aldrich, #238813) and NAC (Sigma-Aldrich #A9165). Recombinant subtilase AB (SubAB) and the inert SubA_A272_B enzyme containing a single amino acid substitution were kind gifts from Drs. Adrienne W Paton and James C. Paton from School of Molecular and Biomedical Science, University of Adelaide, Australia [46].

### Gene expression correlation analyses

Normalized gene expression data were accessed from a previous study [38] through GEO dataset (GSE19276). Hierarchical clustering was performed using Euclidean distance and “Ward.D2” clustering method in R (package “pheatmap”). Heat maps were generated using the heat map function based on a gene set representing common UPR signaling components and enzymes involved in CS polymerization and chain modifications (Supplementary Figs. S3, S4). Correlation analysis assessing the relationship of expression between two genes was generated using Spearman correlation. All analyses were performed in R (version 4.2.0).

### Recombinant protein production

rVAR2 constituting the minimal binding region of VAR2CSA (GenBank accession code GU249598) with a C-terminal V5-tag was produced both in Sf9 cells [84] and in SHuffle T7 Express Competent *E. coli* (NEB) [1], they were purified as previously described. Sf9 cell produced rVAR2 has a Strep tag for purification and the *E. coli* produced rVAR2 has a 6Xhis tag. The monomeric protein was snap-frozen and stored at -80°C until use. Similarly, recombinant DBL4, referred to recombinant control, (non-binding region of VAR2CSA) with a C-terminal V5-tag was produced in Shuffle T7 Competent cells as previously described [1]. Recombinant Chondroitinase ABC (UniProt: P59807), bearing a C-terminal 6×His tag, was expressed in *E. coli* Shuffle T7 Express cells (New England Biolabs, #C3029J). The protein was purified using a two-step chromatography procedure: initial affinity purification on a HisTrap HP column (Cytiva, #17524802), followed by size-exclusion chromatography on a Sephacryl S-300 column (Cytiva # 17116701). The purity and homogeneity of all recombinant proteins were verified by SDS-PAGE.

### rVAR2 immunostaining of tissue

A TMA containing 40 cases (in duplicates) of osteosarcoma from Biomax OS804a Osteosarcoma Tissue Array https://www.biomax.us/OS804 were analyzed for oncofetal CS presentation using immunohistochemistry (IHC) as previously described [1]. Five cores were not evaluable and thus became *n* = 75 (Supplementary Table S1). Briefly, sectioned formalin fixed and paraffin embedded (FFPE) tissue samples were stained with 0.5 nM rVAR2-V5 using the Ventana DISCOVERY Ultra auto-stainer (Ventana Medical Systems, now Roche Tissue Diagnostics) platform, without antigen retrieval, followed by monoclonal anti-V5 step (1:1400) (Invitrogen, #46-0705/#46-070) and anti-mouse-HRP detection (DISCOVERY UltraMap anti-Ms HRP detection kit (Roche, #760-4313). Placental tissue sections with/without chondroitinase ABC (1 U/mL) (Sigma-Aldrich, #C3667) treatment were used as rVAR2 specificity controls. For IHC scoring, values on a four-point scale were assigned to the intensity of each chromogenic immunostaining: descriptively, 0 = no staining, 1 = low, but detectable degree of staining, 2 = clearly positive cases, and 3 = strong reactivity.

### GRP78 and CS-56 immunostaining of human tissue

A TMA containing 41 pediatric sarcoma primary tumors, each of them represented in duplicates, was obtained by the Children’s Oncology Group (COG). The cohort included 19 Ewing sarcoma (46.3%) and 22 osteosarcoma (53.7%) specimens. Additional clinical information about the cohort is reported in Supplementary Table S2. Baked and deparaffinized FFPE sarcoma TMA sections were analyzed for CS (CS-56) and GRP78/BiP expression using the Ventana DISCOVERY Ultra auto-stainer. For CS-56 staining, the tissue section was incubated in Tris-based buffer (CC1, Ventana) at 95 °C for 36 min to retrieve antigenicity, followed by incubation with the primary antibody at RT for 32 min: CS-56 (Abcam, #ab11570) (1:500) diluted in DISCOVERY Ab diluent (Ventana). For GRP78/BiP staining, the tissue section was incubated in Tris-based buffer (CC1, Ventana) at 95 °C for 64 min to retrieve antigenicity, followed by incubation with the primary antibody at RT for 60 min: anti-GRP78/BiP (clone C50B12, Cell Signaling Technology (CST) #3177S), (1:1000) diluted in DISCOVERY Ab diluent (Ventana). Bound primary antibodies were visualized with the DISCOVERY DAB Map Detection Kit (Ventana).

For IHC scoring, values on a four-point scale were assigned to the intensity of each chromogenic immunostaining: descriptively, 0 = no staining, 1 = low, but detectable degree of staining, 2 = clearly positive cases, and 3 = strong reactivity. For CS-56 immunostaining, IHC was quantified for staining intensity (0–3) alone because homogeneous staining was observed throughout the tissue (Supplementary Fig. S2D). For GRP78 immunostaining, IHC was quantified for staining intensity (0 to 3) and percentage of positive cells (0–100%) (Supplementary Fig. S2A). For each sample, the IHC score was calculated as staining intensity multiplied by percentage of positive cells. For IHC data analyses, the average score was calculated for duplicate 1 mm tissue cores from the same patient. Based on the data distribution, the following cut-off points were chosen: for CS-56 immunostaining, the intensity score 1.5 was chosen; for GRP78 immunostaining, H score of 100 was chosen. See Supplementary Fig. S2B, C for antibody specificity controls.

All stained tissue microarray (TMA) slides were digitized with a Leica Aperio AT2 scanner, (Leica Microsystems; Concord, Ontario, Canada) at magnification equivalent to 40X. The images were subsequently stored in the Aperio eSlide Manager (Leica Microsystems) at the Vancouver Prostate Centre. All specimens were examined and scored by pathologist Dr. Htoo Zarni Oo. Wilcoxon signed-rank test was used to compare groups.

### Cell lines and reagents

Human osteosarcoma cell lines were purchased at ATCC or kindly provided by collaborators as indicated (as indicated in the STAR methods). Green fluorescent protein (GFP)-expressing MG63.3 (RRID: CVCL_WL01) was obtained from Dr. Rosandra Kaplan (Pediatric Oncology Branch, NIH, Bethesda, Maryland, USA). MNNG (ATCC® CRL 1547), *e*GFP-expressing MNNG (MNNG^*e*GFP^, derived from (ATCC® CRL 1547)), SJSA-1, OST, MG-63 (ATCC® CRL-1427), HOS (ATCC® CRL-1543) cells and were cultured in HyClone™ Minimum Essential Medium (HyClone, Logan, UT, USA) containing 10% (v/v) fetal bovine serum (FBS) (Gibco, Gaithersburg, MD, USA). Saos-2 (ATCC® HTB-85) cells were grown in McCoy’s 5a Medium Modified (ATCC, MD, USA) supplemented with 10% (v/v) FBS. The isogenic U-2-OS (ATCC® HTB-96) cells (U2-OS^WT^, U2-OS^*CHST11/12*^ and U2-OS^*B4GALT7*^) were grown in Dulbecco’s Modified Eagle Medium (Gibco) supplemented with 10% (v/v) FBS (Gibco). All KO cells for this study were only used for experiments within passage 12 passages. HEK293T (ATCC® CRL-11268) cells were maintained in Dulbecco’s Modified Eagle Medium supplemented with 10% FBS, 2 mM l-glutamine.

All cells were cultured in a humidified chamber at 37 °C, 5% CO_2_ and were ruinously tested free of mycoplasma contamination.

### Generation of U2-OS knock-out cell lines with distinct GAG repertoire

A panel of stable isogenic cell lines based on parental U2-OS cells were generated using targeted gene knock-out with CRISPR/Cas9 gene editing technique to display different repertoires of GAG structures as previously described [85]. The GAG tetra-linker (GlcA-beta1,3-Gal-beta1,3-Gal-beta1,4-Xyl-beta1-*O*-Ser) synthesis was disrupted by knocking out *B4GALT7* encoding the beta-1,4-galactosyltransferase 7, which lays down the first galactose in the tetra saccharide linker [40]. 4-*O*-sulfation of CS was disrupted by knocking out *CHST11* and *CHST12* encoding carbohydrate sulfotransferase 11 and 12 U2-OS^*CHST11/12*^, both of which enzymes catalyze the transfer of sulfate to position 4 of the GalNAc residue of CS [40]. The U2-OS^*CHST11/12*^ cells therefore present less 4-*O*-sulfated CS than U2-OS^WT^ (Supplementary Fig. S6B).

Screening of gRNAs were performed with HEK293T cells as previously described [86, 87]. Briefly, 3 gRNA sequences per gene were designed for *B4GALT7, CHST11*, and *CHST12* genes using the online predictor tool (https://horizondiscovery.com/en/ordering-and-calculation-tools/crispr-design-tool) and were inserted into the gRNA expression plasmid (Addgene plasmid, #68370). HEK293 cells were co-transfected with 1 µg each of gRNA plasmid and GFP-tagged Cas9-PBKS plasmid (Addgene plasmid, #68371) using Lipofectamine 3000 (Invitrogen, #L3000075). Transfected populations were enriched for GFP expression by FACS (SONY SH800, Sony Biotechnology, WA, USA), and a gRNA with highest cutting efficiency was selected by Indel Detection by Amplicon Analysis (IDAA) [88] for further generation of knock-out (KO) cells in U2-OS cells. For generation of the KO, U2-OS cells were seeded at 0.5 ×10^6^ cells/mL in 6 well plates (NUNC, Denmark) one day prior to transfection. Cells were co-transfected with 1 µg each of endotoxin free gRNA (Table 1. List of CRISPR gRNA design and PCR primers) and GFP-tagged Cas9-PBKS plasmid using Lipofectamine 3000. 48 h post transfection, cells were bulk sorted and enriched for (transient) GFP expression by FACS. After culturing for one week, the GFP negative cell pool was further single cell sorted into 96-well plates. Single cell clones were screened by IDAA and selected clones were further verified by Sanger sequencing and HPLC (Supplementary Fig. S6A).

**Table 1:** List of CRISPR gRNA design and PCR primers.

Gene name	gRNA	Forward (5′-3′)	Reverse (3′-5′)
*B4GALT7*	TGACCTGCTCCCTCTCAACG	GGCTGAGTGAAGTCAGTGCT	ACTGCCCCATCCTTCCCA
*CHST11*	GATAAAGGATCCCAAGCAAG	CTCAGCACTTCCAGACCAACTC	TAGGGTTGGAAGAGGCAGAAATC
*CHST12*	CGTGTGCAAGTAGAAGTGCG	CTCTGCAGGAAGCTGAAGTGAG	GTCGTAGCCTCTCACGCTCT

Target gene names, single guide RNA (gRNA) targeting sequences, and corresponding forward and reverse PCR primers (5′→3′) used for genomic validation of knockouts in *B4GALT7*, *CHST11*, and *CHST12* listed in Table 1.

### Cellular rVAR2-V5 binding by flow cytometry

rVAR2-V5 binding (E. Coli produced) was performed as previously described [1]. In brief, osteosarcoma cells were grown to 70–80% confluency before being detached non-enzymatically using Corning CellStripper (Corning, #25-056-CI) and resuspended in FACS buffer (1 X PBS, 2.5 mM EDTA, 2% FBS, 0.05% sodium azide). Cells were then incubated with rVAR2-V5 (2-fold dilutions) ranging from 0 to 200 nM or rDBL4 (200 nM) in round bottom 96-well plates (100,000 cells/well) for 30 min on ice. Competitive inhibition with soluble CSA (400 µg/mL, Sigma Aldrich #27042) served as rVAR2-V5 specificity controls. Cellular rVAR2-V5 binding was detected by anti-V5-FITC (Invitrogen, #R963-25) (1:500) or anti-V5-Alexa 647 (Invitrogen, #451098) (1:250) diluted in FACS buffer, incubating for 30 min on ice. Cells were analyzed (minimum 10,000 (viable) immediately using a FACS Canto II (BD Biosciences) and data weas analyzed using Flowjo v10.1r7 software (Tree Star Inc).

### Cell treatments

For all experiments studying glycocalyx, cells were detached non-enzymatically using Corning CellStripper Dissociating Reagent (Corning #25-056-CI).

To study UPR signaling in MNNG cells, cells were plated 1–2 days in advance (corresponding to 60% confluency at treatment onset of stress), treated for 6 h with 50 nM or 100 nM thapsigargin (TG) (pulse), washed twice in sterile PBS, and cultured in fresh growth media for up to 120 h. Whole cell lysates were obtained (70–80% confluent) for western blotting analysis (see section “SDS-PAGE and western blotting”). Vehicle controls with DMSO were cultured in parallel for all timepoints. Likewise, to study UPR signaling in the U2-OS isogenic cell lines, cells were plated 1–2 day prior to treatments with TN (0.2, 1, or 2 µg/mL), or VEH (DMSO) for 6 h, 8 h or 24 h prior to harvesting whole cell protein lysates (see section “SDS-PAGE and western blotting”). Cell seeding densities were adjusted for 70–80% confluency at harvesting.

To study the effects on ER stress on GAG composition, MNNG cells were detached non-enzymatically and seeded 1–2 days prior to 6 h pulse-treatment with TG (50–100 nM) or TN (50 ng/mL) treatment for 24 h followed by 0–48 h recovery in fresh media for downstream analysis (HPLC, flow cytometry, confocal microscopy). For confocal imaging, cells were grown on coverslips (Azer Scientific Inc, #ES0117520). Cell seeding densities were adjusted for 70–80% confluency at harvesting.

For ER stress live fitness studies in the U2-OS isogenic cell lines, cells were seeded (4000 cells/well) in 96 well plates (~2 h) and treated with TG (25–500 nM) or TN (0.05–2 µg/mL) diluted in growth media containing 250 nM propidium iodide (PI). Vehicle controls (DMSO) were cultured in parallel. Real-time monitoring of cellular fitness is described in section “IncuCyte live cell imaging”.

For chondroitin sulfate A (CSA) rescue assays, U2-OS^WT^ and U2-OS^*B4GALT7*^ cells were ± pre-treated for 24 h in sterile filtered chondroitin sulfate A (CSA, 100 mg/mL, average MW ~ 20 kDa) (Sigma-Aldrich, #27042), seeded at 4000 cells/well in 96 well plates (~2 h) prior to TG (5–500 nM) or vehicle in the continued presence of CSA (100 mg/mL). Sterile propidium iodide was added at 250 nM and allowed for real-time monitoring of cellular fitness as described in section “IncuCyte live cell imaging”.

For pan-caspase inhibition experiments, U2-OS isogenic cell lines were seeded (4000 cells/well) in 96 well plates and upon full attachment treated with Z-VAD-FMK (10 µM) or Q-VD-OPh (20 µM) ± TG (200 nM), ±TN (1 µg/mL) or vehicle (DMSO) diluted in growth media. Cells were treated with caspase inhibitors 1–2 h prior to pharmacologically inducing ER stress. Sterile propidium iodide was added at 250 nM and allowed for real-time monitoring of cellular fitness as described in section “IncuCyte live cell imaging”.

For necroptosis and ferroptosis inhibition experiments, U2-OS isogenic cell lines were seeded (4000 cells/well) in 96 well plates and upon full attachment treated with NST (30 uM) or FST (2 µM) ± TG (200 nM), ±TN (1 µg/mL) or vehicle (DMSO) diluted in growth media. Cells were treated with NST or FST to block, necroptosis or ferroptosis, respectively, 1–2 h prior to pharmacologically inducing ER stress. Sterile propidium iodide was added at 250 nM and allowed for real-time monitoring of cellular fitness as described in section “IncuCyte live cell imaging”.

For apoptosis assessment (caspase 3 cleavage and Annexin V/propidium iodide (PI)) in U2-OS isogenic cell lines, cells were plated (100,000 cells/well) in 6 well plates and treated with TG (200 nM) or vehicle (DMSO) for 32 h prior to performing the Annexin V-FITC/PI apoptosis assay (section “Annexin V-FITC/PI apoptosis assay”).

For site-specific degradation of GRP78, the U2-OS isogenic cell lines were seeded (4000 cells/well) in 96 well plates and treated with subtilase AB (subAB) or the inert SubA_A272_B variant (100 ng/mL) for 3 h prior to TG (50 nM) co-treatment. Vehicle controls (DMSO + glycerol) were cultured in parallel. Sterile propidium iodide was added at 250 nM and allowed for real-time monitoring of cellular fitness as described in section “IncuCyte live cell imaging”.

### GAG disaccharide analysis by HPLC

After ended treatment, MNNG cells were collected non-enzymatically using Corning CellStripper Dissociating Reagent (Corning, #25-056-CI) at 80% confluence. Cells were pelleted by centrifugation and washed in ice-cold PBS and lysed in EBC buffer (50 mM Tris-HCl (pH 7.4), 120 mM NaCl, 0.5% IGEPAL CA-630, 1 mM EDTA, 2.5 mM MgCl_2_) on ice. Lysates were frozen (−80 °C), thawed on ice and protein content was determined by Pierce BCA Protein Assay Kit (Thermo Fisher #A65453). The cell extracts were digested in 0.5 M CaCl_2_, 0.1% Triton X-100 and 1 mg/mL Pronase E (protease from *Streptomyces griseus*, Sigma-Aldrich, #P8811) and incubated overnight at 37 °C on a shaker. The samples were heat-inactivated at 100 °C for 10 min and allowed to cool before the addition of 20 kU/mL DNase (Deoxyribonuclease I from bovine pancreas, Roche, #11284932001). After 2 h incubation at 37 °C, the cell extracts were centrifuged at 16,000 × *g* for 10 min and the pellets were discarded. 0.5 mL DEAE sepharose (DEAE-Sephacel, GE healthcare) was equilibrated in a disposable column (2 mL bed volume Poly-Prep® Chromatography Columns, Bio-Rad) with 20 mL equilibration buffer (50 mM NaOAc, 0.2 M NaCl, 0.1% Triton X-100, pH 6.0). The cell extract was mixed with 10 mL equilibration buffer and loaded onto the column. The column was then washed with 20 mL wash buffer (50 mM NaOAc, 0.2 M NaCl, pH 6.0) and eluded with 2.5 mL elution buffer (50 mM NaOAc, 1.0 M NaCl, pH 6.0). The eluted samples were divided into 1.7 mL Eppendorf tubes and ice-cold ethanol saturated with NaOAc (1:3, v:v) was added to a final concentration of 70%. The samples were precipitated at −20 °C overnight and then centrifuged at 20,000 × *g* and 4 °C for 20 min. Following precipitation, the ethanol was discarded, and the GAG samples were speed-vacuumed until dry. For CS analysis, the dried GAGs were resuspended in 100 μl ChABC buffer (50 mM Tris, 50 mM NaCl, pH 7.9) and digested with 100 mU/mL ChABC (Chondroitinase ABC from *Proteus vulgaris*, Sigma-Aldrich, #C3667) for 2 h at 37 °C. For HS analysis, the GAGs were resuspended in 100 μl heparinase buffer (40 mM ammonium acetate and 3.3 mM calcium acetate, pH 7.0) and digested with 20 mU/mL heparinase I-III (Iduron #HEP-ENZ I-III). The samples speed-vacuumed until dry and were analyzed as described by Volpi et al. [89]. Briefly, the samples were dissolved in 5 μL 0.1 M AMAC (2-aminoacridone, Sigma-Aldrich, #06627) in glacial acetic acid-DSMO (3:17, v:v) and incubated for 15 min at RT. The samples were then mixed with 5 μL 1 M NaCNBH_3_ and incubated for 3 h at 45 °C. To remove excess AMAC, the samples were precipitated in acetone. Labeled disaccharides were analyzed using a BEH C18 (1.7 µm, 2.1 ×150 mm) column, running on a Waters Acquity UPLC system with a fluorescence detector. CS/DS disaccharides were separated with 80 mM ammonium acetate as mobile phase A (pH 5.5) and HS disaccharides with 150 mM ammonium acetate as mobile phase A (pH 5.6). 100% acetonitrile was used as mobile phase B for both CS/DS and HS. For separation, a gradient of mobile phase B increasing from 3 to 13% over 30 min at a flow rate of 0.2 mL/min was used. Labeled GAGs were quantified (Area Under the Curve) using a series of disaccharide standards (20 pmol AMAC-labeled disaccharides; Iduron). The total CS amount was normalized to total protein content, as determined by the BCA assay. Disaccharide abundance, specifically dp2, was then expressed relative to the total CS content to account for variations in overall CS levels across samples.

### Radiolabeling and CS size analysis

After ended treatment, MNNG cells were preconditioned in MEM medium without FBS and incubated for 30 min at 37 °C. The samples were then radiolabeled with [^35^S] sulfate (50 µCi/mL) in MEM media containing 10% dialyzed FBS for 48 h at 37 °C. The cells were then detached non-enzymatically and treated with trypsin to allow the collection of cell surface GAGs. CS purification was performed as described in the section Disaccharide analysis by HPLC. The samples were fractioned on size exclusion chromatography using a Sepharose 6B-L column (1 × 70 cm) (Cytivia, #17016001) equilibrated in running buffer (50 mM sodium acetate, 0.2 M NaCl, pH 6.0). The [^35^S]CS samples were split in two, added to the column and fractions of 1.25 mL were collected. Lastly, sample fractions were mixed with 5 mL of Ultima Gold XR scintillation fluid (PerkinElmer Life Sciences) and analyzed using a scintillation counter. Each dataset was fitted to two main populations using a Chi-square statistical model and the approximate molecular weight distributions were calculated as described in ref. [90].

### GAG detection by flow cytometry

After ended treatment, MNNG cells were detached non-enzymatically using Corning CellStripper (#25-056-CI) and stained in a round bottom 96-well plate (100,000 cells/well). A portion of the cells were pre-treated using HSases (5mU/mL heparin lyases II (IBEX, #60-018) and III (IBEX # #60-020), ChABC (20 mU/mL, Sigma Aldrich, #C3667) for 30 min at 37 °C in dPBS to digest GAG chains and create stub neoepitopes. HS Chains, CS chain, and proteoglycan levels were assessed using primary antibodies: anti-HS stub mAb (1:200) (clone F69-3G10, AMSBio, #370260-S), anti-HS mAb (1:1000) (clone F58-10E4, AmsBio #370255-1), anti-C4S “stub” mAb (1:200) (clone 2B6, Cosmo Bio, #CAC-PRPG-BC-M02) or anti-CS (CS-56) mAb (1:500) (AMSBio, #270695) diluted in 2% FBS in dPBS (PBS2) on ice for 30 min. 2B6 and F69-3G10 binding was detected using anti-mouse IgG-Alexa488 (1:1000) (Invitrogen, #A28175) while CS-56 and F58-10E4 were detected by goat anti-mouse IgG polyclonal Alexa 488 Antibody (1:1000) (Invitrogen, #A-11001). Oncofetal CS was detected using pre-conjugated rVAR2-SpyTag:SpyCatcher-Alexa647 (50 nM). For this, the SpyCatcher was conjugated with Alexa Fluor^TM^ 647-NHS (Invitrogen, #A20006) in a 1:3 molar ratio and prepared according to the manufacturer’s protocol. Cells were analyzed (minimum 10,000 (viable) cells) using a FACSCalibur (BD Biosciences) flow cytometer. Data was analyzed in FlowJo.

### Oncofetal chondroitin sulfate visualization by confocal microscopy

MNNG cells exposed to a 6 h TG or vehicle (DMSO) pulse as described previously recovered in growth media for 24 h or 48 h on glass coverslips (Azer Scientific Inc, #ESO117520). Cells were fixed in 4% paraformaldehyde (PFA) (Santa Cruz, #sc-281692), permeabilized in perm buffer (1xPBS, 0.1% triton-X100) for 2 min, blocked in blocking buffer (3% BSA, tris-buffered saline, 0.05% tween20) for 1 h, and stained for oncofetal CS and Golgi apparatus using rVAR2-V5 (50 nM) and anti-58K Golgi protein (1:300) (clone 58K-9, Abcam, #ab27043), respectively, overnight at 4 °C in a humidified chamber. rVAR2-V5 was detected with anti-V5 (SV5-Pk1) (Invitrogen, #R960CUS) (1:500) and anti-Rabbit-Alexa488 (1:300) (Invitrogen, #R37118). 58K-Golgi antibodies were detected with anti-mouse-Alexa568 (1:300) (Invitrogen, #A-11031). To stain oncofetal CS in the cellular glycocalyx, live cells were treated with rVAR2-V5 (100 nM) for 15 min at 37 °C prior to fixation and visualization by anti-V5 (Invitrogen, #R960CUS) (1:500) and anti-Rabbit-Alexa488 (1:300) (Invitrogen, #R37118). Nuclei were stained with DAPI (Invitrogen, #D3571). Slides were analyzed by laser-scanning confocal microscopy (Zeiss LSM780 (Oberkochen, Germany) and Olympus Scientific’s FV3000 (Tokyo, Japan)).

### Crystal violet viability assay

MNNG cells were grown for 3 days in the presence of rVAR2 (0, 10, or 20 nM), fixed in 1% glutaraldehyde solution (Sigma Aldrich, #354400) for 10 min. After the fixation, the cells were washed with water and stained with 0.1% crystal violet solution (Sigma Aldrich, #C0775) at RT for 30 min on a shaker as previously described [91]. Cells were subsequently rinsed in tap water to remove non-bound dye. After air drying, bound crystal violet dye was dissolved completely in Sorenson’s solution and absorbance (560 nm wavelength) was measured on a BioTek Gen5 microplate reader.

### IncuCyte live cell imaging

Cellular PI uptake (red area) and phase contrast images (10X magnification) were obtained for death and confluence assessment, respectively. Cell death and confluence were monitored continuedly every 4 h for up to 72 h using the IncuCyte S3 Live-cell analysis system (Essen BioScience, Michigan, USA) and analyzed in InCuCyte Software (v2020C). Death is reported as total red area ×1000 µm ^2^/well or total red area ×1000 µm^2^/well normalized to time 0 if cell death was present at onset of experiment due to some transfection induced toxicity.

### Glycocalyx oncofetal CS levels by western blotting

Binding of rVAR2-V5-his to MNNG and MG-63 cells was also determined by western blotting. MNNG cells exposed to a 6 h TG or vehicle (DMSO) pulse as described previously and recovered in growth media for up to 48 h were incubated with rVAR2-V5-His (100 nM) in the presence of absence of soluble CSA (400 µg/mL) diluted in growth media for 15 min at 37 °C prior to harvesting. Likewise, MG-63 cells exposed to TG (0.5 µM or 1 µM), tunicamycin (TN) (2 µg/mL or 4 µg/mL) or vehicle (DMSO) for 24 h, were incubated with rVAR2-V5-His in the presence of absence of soluble CSA as described above. All cells were washed twice in ice-cold PBS to remove unbound protein and whole cell lysates were obtained, see section “SDS-PAGE and western blotting”.

### SDS-PAGE and western blotting

Whole cell extracts were obtained by lysis of 70–80% confluent cells. Briefly, cells were washed with ice-cold PBS before on-plate lysis with EBC lysis buffer (50 mM Tris-HCl (pH 7.4), 120 mM NaCl, 0.5% IGEPAL CA-630, 1 mM EDTA, 2.5 mM MgCl_2_) supplemented with 1× cOmplete^TM^ protease inhibitor cocktail (Roche, #04693116001) and PhosSTOP^TM^ phosphatase inhibitors (Roche, #04906837001) or RIPA lysis and extraction buffer (Thermo Scientific, #89901) supplemented with 1× cOmplete protease inhibitor cocktail and phosSTOP phosphatase inhibitors. Cell lysis occurred on ice for 15–30 min and then lysates were frozen for min 30 min at −80 °C. Lysates were centrifuged at 13,000 × *g* for 10 min at 4 °C to remove cell debris and total protein concentration was measured using the Pierce BCA protein Assay Kit (Thermo Scientific, #A65453) according to manufacturer’s guide. For analysis, equivalent amounts of total protein (10–50 µg/well) were subjected to SDS-PAGE separation in 10-well or 15-well 4–15% Mini-PROTEAN® TGX™ Precast Protein Gels (Bio-Rad #4561084, #4561085, and #4561086) under reducing conditions. Resolved proteins were blotted onto 0.2 µm nitrocellulose membranes (Bio-Rad) using Bio-Rad’s Trans-Blot Turbo system, and blocked in 5% milk or 5% BSA (antibody-specific) in Tris-Buffered Saline and Tween 20 (TBS-T, 0.05%) for 1 h at RT. The membranes were incubated overnight with primary antibodies (anti-vinculin (1:4000) (clone VIN-54, #ab130007), anti-GRP78/BiP (1:1000) (C50B12, #3177S), anti-GAPDH (1:1000) (clone D16H11, CST #5174S, anti-V5-Tag (1:1000) (clone E9H80, CST, #80076), anti-CHST11 (1:500) (Sigma Aldrich #WH0050515M1), anti-ATF4 (1:1000) (clone D4B8, Cell Signaling Technologies #11815S), anti-Ire1α (1:1000) (clone 14C10, #3294S), Perk (1:1000) (Clone D11A8, Cell Signaling Technologies #5683S), Phospho eIF2α (Ser51) (1:1000) (Cell Signaling Technologies, #9721S), anti-Eif2α (1:1000) (Cell Signaling Technologies, #9722S), XBP-1s (1:1000) (clone D2C1F), Cell Signaling Technologies #12782S) diluted in 2.5% blocking solution (Milk or BSA) at 4 °C, washed in TBS-T, and detection of immune complexes was done using one of two methods (i) with corresponding horseradish peroxidase (HRP) labeled species specific antibodies (anti-rabbit IgG HRP (cell signaling technology, #CS7074S), anti-mouse IgG HRP (cell signaling technology, #7076S), and anti-His HRP (Miltenyi Biotec, #130-092-783, Bergisch Gladbach, Germany); or (ii) with corresponding fluorophore labeled species specific antibodies purchased from LI-COR (donkey anti-mouse IgG (H + L) IRDye® 680RD (#926-68072), donkey anti-rabbit 800CW conjugated (#926-32213), donkey anti-mouse IgG antibody, 800CW Conjugated (#926-32212), donkey anti-rabbit IgG Antibody, 680RD conjugated (#926-68073)). Detection of immune complexes was done using (i) SuperSignal West Femto Maximum Sensitivity Substrate (Thermo Scientific #34094) according to manufacturer’s instruction and images were captured with a G:Box XT4 Chemiluminescence and Fluorescence Imaging System (Syngene, Cambridge, UK) or by (ii) scanning the membranes using the LI-COR Odyssey® Gel Imaging system.

### Quantitative real-time PCR

After the treatments ended, RNA was extracted from MNNG cells using the Monarch® Total RNA Miniprep Kit (New England Biolabs #T2010S) as per manufacturer’s protocol. Purified RNA was quantified using ThemoFisher’s NanoDrop Spectrophotometer and 1 µg RNA was reverse-transcribed into cDNA using the LunaScript® RT SuperMix Kit (New England Biolabs, #E3010L) in a 20 µL reaction according to manufacturer’s protocol (reaction: 2 min at 25 °C, 10 min at 55 °C, 1 min at 95 °C). The resulting cDNA was diluted and amplified in a 10 µL reaction using Luna® Universal qPCR Master Mix (New England Biolabs #M3003L) and gene-specific forward and reverse primers (0.6 µM) for 45 cycles in the ViiA™ 7 Real-Time PCR system (Applied biosystems, Thermo Fisher) according to manufacturer’s protocol.

Gene expression levels of *HSPA5* were normalized to S18 and RPL32 expression levels and presented relative to vehicle treated controls using the ViiA^TM^ software. A list of primers used in the study can be found in section “Star methods”.

### Analysis of sGRP78

After ended TG/DMSO (100 nM) treatment, secreted GRP78 (sGRP78) was collected and analyzed in isogenic U2-OS cell lines (see section “Cell Treatment section”). Conditioned media was collected after ~17 h treatment (under serum starvation), centrifuged at 400 × *g* at 4 °C for 5 min to remove cell debris. Supernatants were transferred into Pall Microsep Advance Centrifugal Devices with Omega Membrane (MWCO 30 K) (Fisher Scientific #MCP030C41) and centrifuged at 3220 × *g*, at 4 °C for 90 min. The concentrate was then collected immediately and stored at −80 °C. Corresponding whole cell lysates were collected in RIPA buffer and used to normalize GRP78 levels to total protein content. GRP78 levels in lysate (10 µg protein/well) and media (37.5 µL) were determined by SDS-PAGE and western blotting. Recombinant human GRP78 (50 ng) was used as positive antibody control for media samples.

### Proteolytic inhibition

Isogenic cell lines were seeded 3 days prior to the proteolytic inhibition experiment in T75 flasks. Cells were then treated with MG-132 (5 μM) in combination with TG (100 nM). Single treated and vehicle controls (DMSO) were cultured in parallel. Whole lysates were obtained after 8 h as described in section “SDS-PAGE and western blotting” and GRP78 expression was assessed. Anti-pan-ubiquitin blotting verified proteasome inhibition.

### Cycloheximide chase assay

For cycloheximide treatment, all U2-OS isogenic cells were grown to ~60%? confluence in 10 cm plates, followed by 0 h, 3 h, 6 h, 24 h treatment with 50 μg/ml cycloheximide (Selleck Chemicals, #S7418) in combination with TG (100 nM), TN (1 μg/ml), or vehicle (DMSO). Cells were washed with cold PBS, then lysed and harvested.

### CHST11 gene overexpression

U2-OS^WT^ and U2-OS^*CHST11/12*−/−^ cells were seeded (100,000 cells/well) in 6 well plates (Corning) one day prior to transfection. Lipofectamine 3000 (Invitrogen, #L3000075) was used in accordance with the manufacturer’s protocol using 1 µg pCMV3-C-His Negative Control Vector (C-terminal His-tagged) (Sino Biological #CV015) (referred to as pVector) or 1 µg pCMV3-human CHST11/C4ST-1 transcript variant 2 Gene ORF-C-His (C-terminal His-tagged) (Sino Biological #HG11396-CH) (pCHST11) and Gibco’s OptiMEM media (Gibco #11058-021). At 48 h, the cells were lifted using Corning CellStripper Dissociating Reagent, re-plated in Corning’s 96 well plates (6000 cells/well) in the presence of PI (250 nM), and 2 h later subjected to pharmacological ER stressors (TG (200 nM) and TN (1 µg/mL)) or vehicle (DMSO) under continuous monitoring (4 h intervals) by the InCuCyte S3 LiveCell system as described in the section “IncuCyte live cell imaging”. CHST11 protein expression was verified at 72 h post transfection using SDS-PAGE and Western Blotting. If transient transfection efficiency was deemed insufficient based on predefined experimental quality criteria, the corresponding downstream analyses were excluded from the biological replicates.

### *Anoikis* in ultra-low attachment plates

The isogenic U2-OS cell lines lifted by Corning CellStripper Dissociating Reagent were seeded (150,000 cells/well) in Costar ultra-low attachment 6-well plates (Corning, #3471) and *anoikis* was assessed after 15 h culture (see sections “Active caspase 3 assessment by flow cytometry” and “Annexin V-FITC/PI apoptosis Assay”).

### Soft agar growth assay

Colony formation in soft agar was assessed similar to previously described [92]. Briefly, two layers of agarose containing DMEM media and 10% FBS were used in this assay, each layer consisting of 1.5 mL in 6-well plates. The top layer and bottom layer contained 0.25% and 0.4% agarose, respectively. U2-OS isogenic cell lines were detached in Corning CellStripper Dissociating Reagent and plated (50,000 cells/top layer), while the bottom layer was cell free. To prevent cells from drying out, 1 mL of DMEM media was added on top of the top layer. Only the top layer and the feeder media contained the antioxidant treatments, i.e. N-acetyl cysteine (NAC) (5 mM) or Trolox (25 mM). The cells were grown for 3 weeks, and live colonies were stained with MTT (0.5 mg/mL) (Aigma-Aldrich, #M2128) for 3 h. Images were then taken using EVOS XL Core microscope using 20x magnification.

### Annexin V-FITC/PI apoptosis assay

Annexin V-FITC early apoptosis detection assay was performed according to the manufacturer’s recommendations (CST, #6592S). Briefly, after treatment ended, adherent U2-OS isogenic cell lines were trypsinized and collected in growth media and were combined with floating cells by centrifugation (300 × *g* at 4 °C, 5 min). Cells cultured in low attachment plates were collected by centrifugation (300 × *g* at 4 °C, 5 min). Cells were washed in ice-cold PBS and then resuspended in 1× Binding buffer provided in the kit. Finally, 96 µL cell suspension (1 × 10^6^ cells) then reacted with 4 µL of annexin V-FITC reagent and 12.5 µL of PI solution for 10 min on ice in the dark. Stained cells (minimum 10,000 events) were analyzed immediately by flow cytometry (FACSCanto^TM^ II, BD Bioscience) and data was analyzed in FlowJo v10.1r7.

### Active caspase 3 assessment by flow cytometry

After ended treatment, adherent U2-OS isogenic cell lines were collected by trypsinization (Thermo Fisher, #25200056) in growth media or simple centrifugation (when grown in low attachment plates), washed in ice-cold FACS buffer (1 × PBS, 2.5 mM EDTA, 2% FBS, 0.05% sodium azide), then cells were fixed in 4% PFA paraformaldehyde (Santa Cruz Bio, #sc-281692) at RT for 20 min, washed in FACS buffer, permeabilized in Perm buffer (1 × PBS, 0.1% saponin, 2.5 mM EDTA, 2% FBS, 0.05% sodium azide) for 10 min prior to staining with V450 anti-active caspase 3 (BD Biosciences, #560627) (1:20) in Perm buffer for 45 min in the dark. Finally, the cells were washed and analyzed (minimum 10,000 events) by flow cytometry (FACSCanto^TM^ II, BD Bioscience) and data were analyzed in FlowJo v10.1r7.

### Ex vivo pulmonary metastasis assay (PuMA)

For the ex vivo PuMA experiments, a pilot study and prior experience with the model were used to guide sample size selection. Specifically, effect size estimates obtained from a pilot experiment (Supplementary Fig. S17) informed the number of lung tissue samples included in subsequent biological experiments. All ex vivo animal studies were conducted in accordance with the University of British Columbia Animal Care Committee regulations (Protocol #A17-0002). The PuMA was performed as previously described [52]. Briefly, female nude mice (NOD.Cg-Prkdcscid/J) aged 6–8 weeks were injected i.v. through tail vein with 400,000 *e*GFP expressing MNNG (highly metastatic osteosarcoma cell line). Before injection, cells were incubated with rVAR2 (400 nM, 30 min at 4 °C), chondrotinase ABC (Sigma, #C2905) (0.4 mg/mL, 1 h at 37 °C) or vehicle (PBS). Then, cells were washed twice with cold PBS (Dulbecco’s Phosphate Buffered Saline, Sigma Aldrich, #D8537) and resuspended in 100 mL of PBS. Fifteen minutes post injection, mice were euthanized using CO_2_, and the trachea was cannulated with a 21G intravenous catheter and attached to a gravity perfusion apparatus. The lungs were filled in the vertical position with heated agarose medium solution containing M-199 media, sodium bicarbonate, hydrocortisone, bovine insulin, penicillin/streptomycin (pen/strep) and agarose. After tying off the trachea with suture, lungs were extracted and placed in sterile PBS with pen/strep on ice for 1 h. Lungs were cut into ~3 mm × 1.5 mm sections using sterile forceps and microdissection scissors. Lung section derived from different mice were distributed across experimental groups to minimize potential bias associated with individual animals. Lung sections were cultured on media-saturated Gelfoam® using 10 lung sections per treatment condition. Media was changed every 48 h and lung slices were imaged with a fluorescent microscope (Axio Observer Z1; Carl Zeiss, Germany) at days 0, 3, 7, and 14. All images were captured using a 5× objective lens and at identical exposure times. The signal intensities were analyzed using ImageJ software. No blinding was performed during the experiments or outcome assessment. No animals were excluded from the analyses. Animal inclusion criteria were predefined and consisted of healthy mice meeting the experimental requirements.

### *PuMA* tissue immunostaining

FFPE MG63.3 PuMA lungs were obtained from a previous study [93] were sectioned in series. Slides were baked at 65 °C for 30 min to melt the paraffin, followed by deparaffinization through sequential incubations in HistoChoice ^®^ Clearing Agent (Sigma Aldrich, #H2779), absolute >99.8% ethanol (VWR, #20821.310), 96% ethanol (VWR, #20824.365), 70% ethanol (VWR, #83801.290), and water.

One slide was subjected to GRP78 immunostaining. Heat-induced antigen retrieval (HIER) was performed in 10 mM sodium citrate (pH 6) for 20 min at boiling temperature. After cooling, slides were blocked for 1 h at room temperature (RT) in 1×PBS supplemented with 5% fetal bovine serum (FBS, Gibco^TM^, #10270-106) and 1% bovine serum albumin (BSA, Sigma Aldrich, # A3059), followed by permeabilization for 15 min RT in PBS, 0.1% Triton X-100. After three washes in PBS, sections were incubated overnight at 4 °C with anti-GRP78/BiP mAb (Cell Signaling Technology, #3177S; clone C50B12) diluted 1:200 in the antibody buffer (PBS, 0.25% BSA). Slides were washed three times and incubated for 1 h, RT with anti-rabbit IgG (H + L)-Alexa Fluor^TM^ 647 (Invitrogen, Cat#A-21245, RRID: AB_2535813) diluted 1:100 in the antibody buffer. Following two PBS washes, cell nuclei were counterstained with 5 μg/ml of 4′,6-diamidino-2-phenylindole (DAPI, Invitrogen, #D1306) for 5 min, RT. Slides were last rinsed in ultra-pure water, dried and mounted using an aqueous mounting media (Dako, #33025).

Two consecutives slides were subjected to oncofetal-chondroitin sulfate staining either with or without chondroitinase ABC treatment as specificity control. The staining was performed as in ref. [37] using the F8 scFv^2^ construct.

Hematoxylin and eosin (H&E) staining was performed on a last section for morphological assessment. Rehydrated slides were incubated for 3 min in Mayer’s hematoxylin (Merck, #MHS32), rinsed in warm running tap water for 15 min, and briefly immersed in distilled water (30 s) and 96% ethanol (30 s). Sections were then stained in eosin (Sigma-Aldrich, #HT110132) for 3 min. Tissues were dehydrated through solutions in ascending concentrations (from 70% ethanol, to the HistoChoice solution) mirroring the deparaffinization in reverse order. Slides were dried, and mounted using VectaMount Express Mounting medium (Vector Laboratories, #H-5700-60).

Imaging was done on automated slide scanners (×20 magnification, 0.8 NA objective): Zeiss Axio Z1 (for H&E-stained sections) or Zeiss Axioscan 7 (for GRP78/ofCS stained sections). Image acquisition and analysis were conducted on ZEN blue software (Zeiss).

### Statistics

Where possible, pilot experiments were performed to estimate effect sizes. These estimates were subsequently used for power calculations. For in vitro experiments with technical replicates, experiments were independently repeated at least three times to ensure reproducibility. Data distribution was assumed to be normal based on prior experience with the assays and the use of technical and biological replicates where sample sizes were small. Variance was assumed to be similar between groups for parametric analyses. For analyses of tissue staining’s, non-parametric statistics were applied using the Wilcoxon signed-rank test for group comparisons. Variation within each group is indicated in the figures and figure legends using standard deviation (SD) or standard error of the mean (SEM), as specified for each experiment. In addition to what is described under ‘Radiolabeling and CS size analysis’, ‘Gene expression correlation analyses’, the statistical analyses were performed using GraphPad Prism (GraphPad Software, CA, USA). For datasets involving two independent variables, two-way ANOVA followed by Sidak’s, Dunnett’s or Turkey’s multiple comparisons tests was used to assess differences among group means. When analyzing data with a single independent variable and three or more groups, one-way ANOVA was used, followed by appropriate post hoc tests. For comparisons between two groups only, a Student’s *t* test (unpaired) was applied.

## Supplementary information

Supplementary Figure S1

Supplementary Figure S2

Supplementary Figure S3

Supplementary Figure S4

Supplementary Figure S5

Supplementary Figure S6

Supplementary Figure S7

Supplementary Figure S8

Supplementary Figure S9

Supplementary Figure S10

Supplementary Figure S11

Supplementary Figure S12

Supplementary Figure S13

Supplementary Figure S14

Supplementary Figure S15

Supplementary Figure S16

Supplementary Figure S17

STAR Methods

Supplementary Figure Lengends

Table S1

Table S2

Original Data

## Data Availability

All data needed to evaluate the conclusions in the paper are present in the paper and/or the Supplementary Materials.

## Code availability

No custom code central to the conclusions of this study was generated. Hierarchical clustering analyses were performed in R (version 4.2.0) using publicly available functions from the pheatmap package with Euclidean distance and the Ward.D2 clustering method. Code used for data analysis is available from the corresponding author upon reasonable request.
