# Supplementary material for: Chondroitin sulfate shapes the unfolded protein response in osteosarcoma to sustain cancer cell survival during ER stress
Source: Cell Death Dis. 2026 Aug 1;17(1):840. doi: 10.1038/s41419-026-09151-9 (PMC13620160; doi:10.1038/s41419-026-09151-9)
Supplement: Supplementary file 1 [file 41419_2026_9151_MOESM22_ESM.pdf]

# Figure 2 uncropped western blots

## For 2E

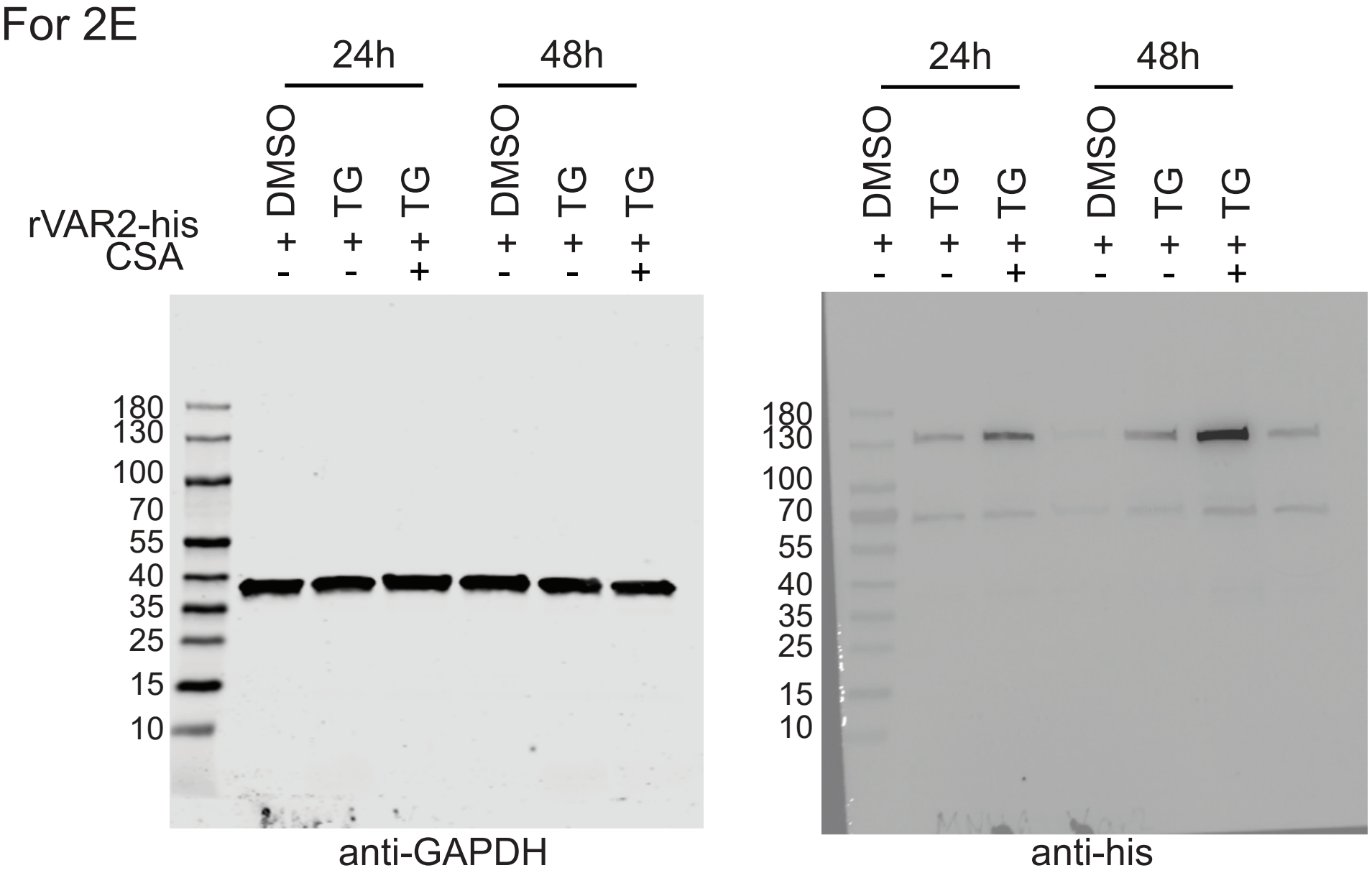


## For 2F

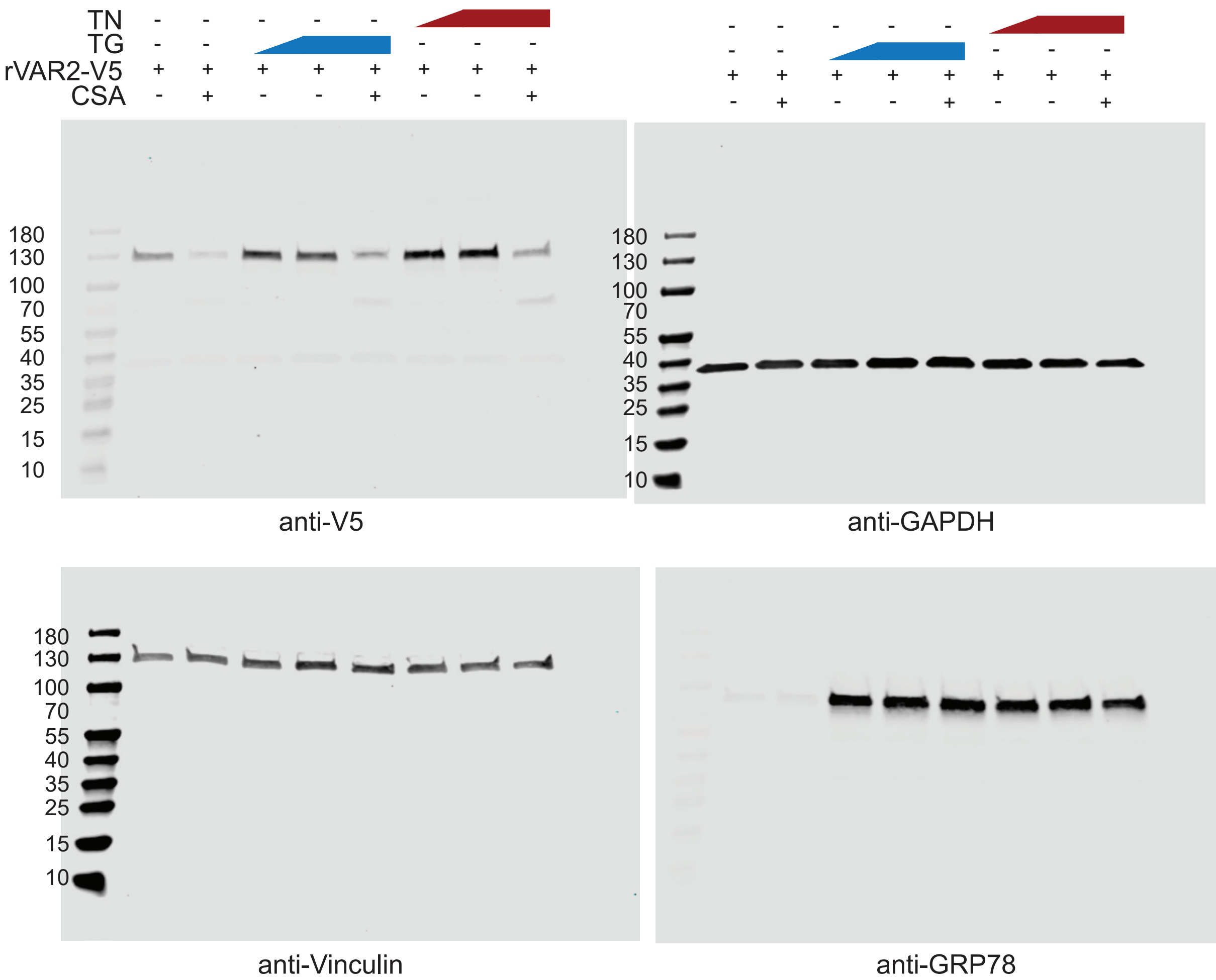

# Figure 3 uncropped western blots

For 3B

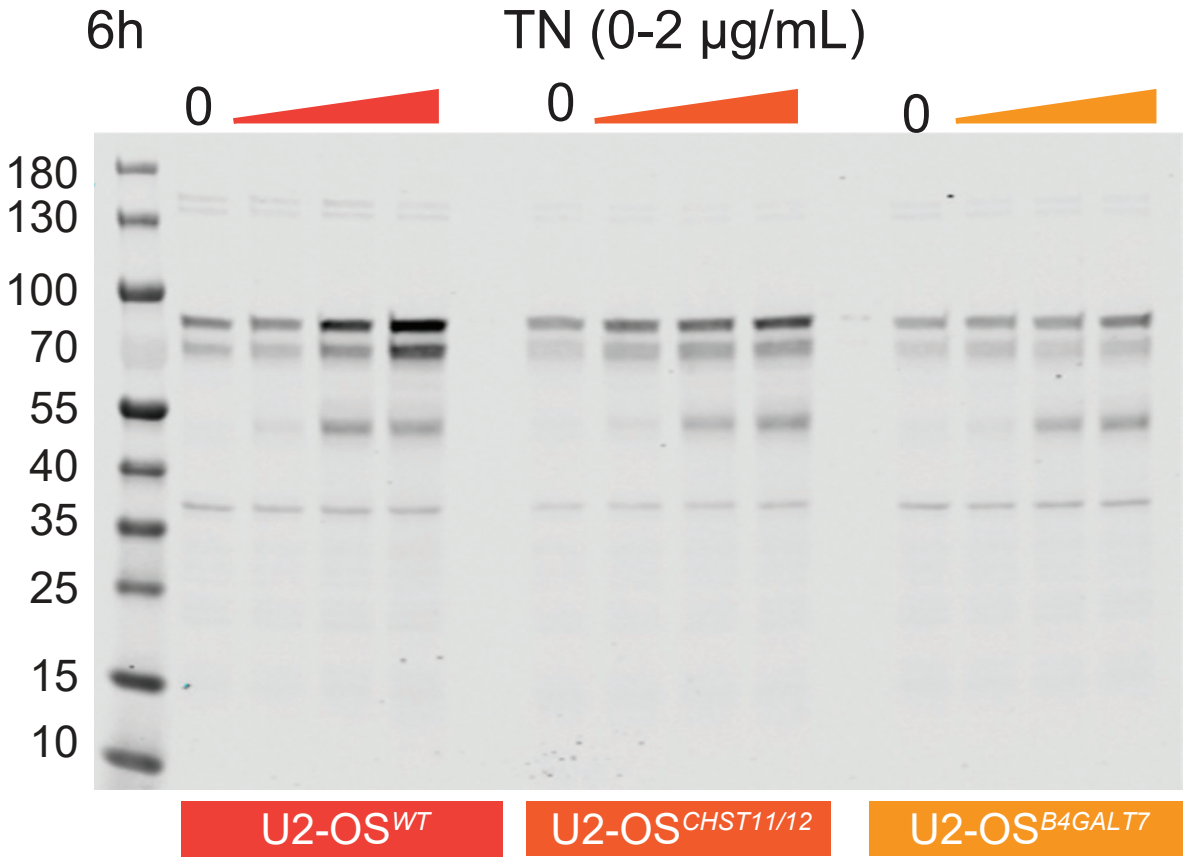


anti-GRP78 + anti-ATF4

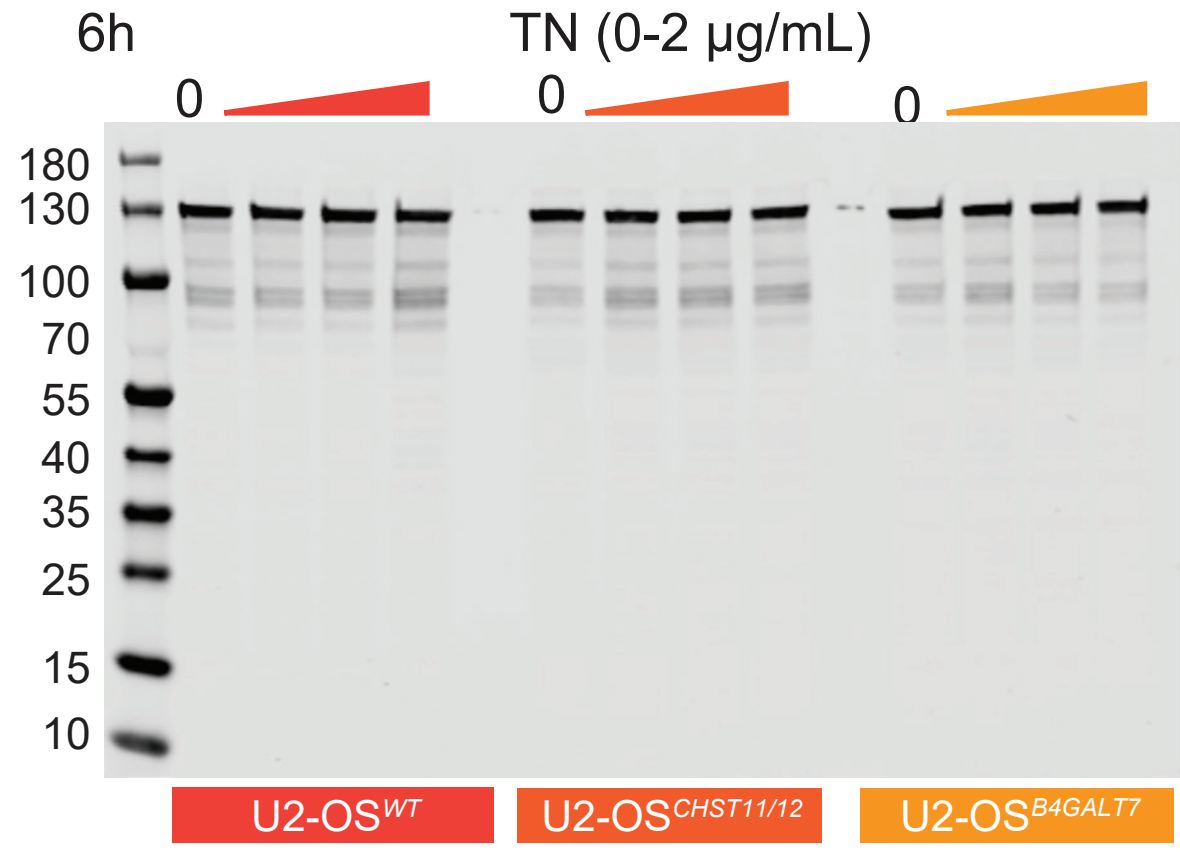


anti-vinculin

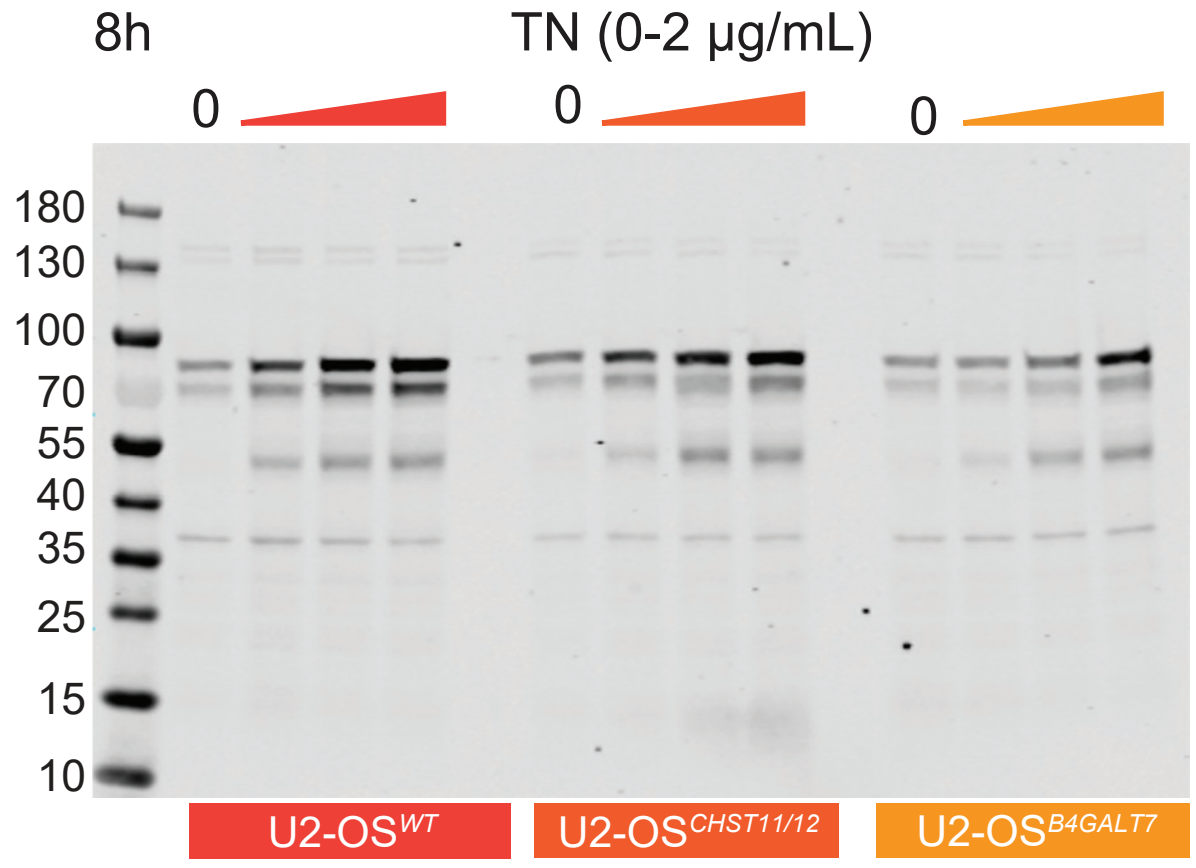


anti-GRP78 + anti-ATF4

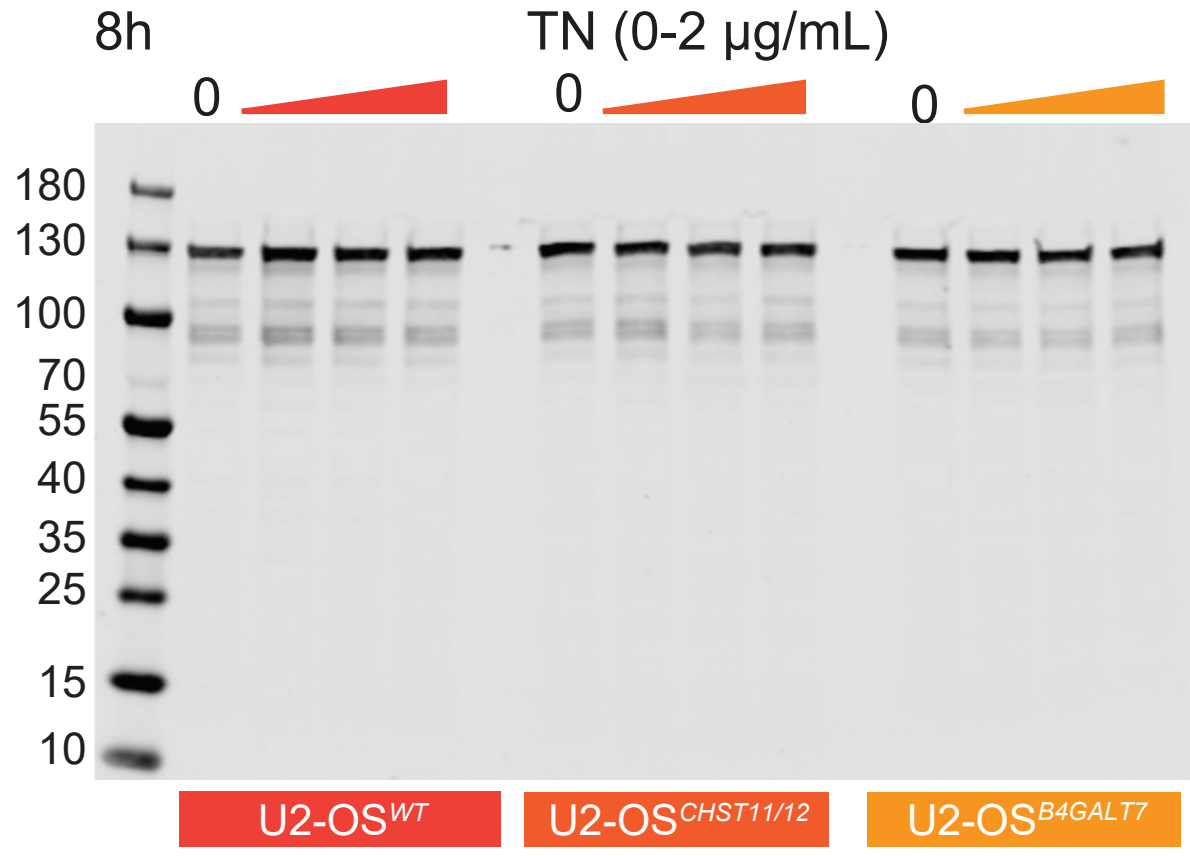


anti-vinculin

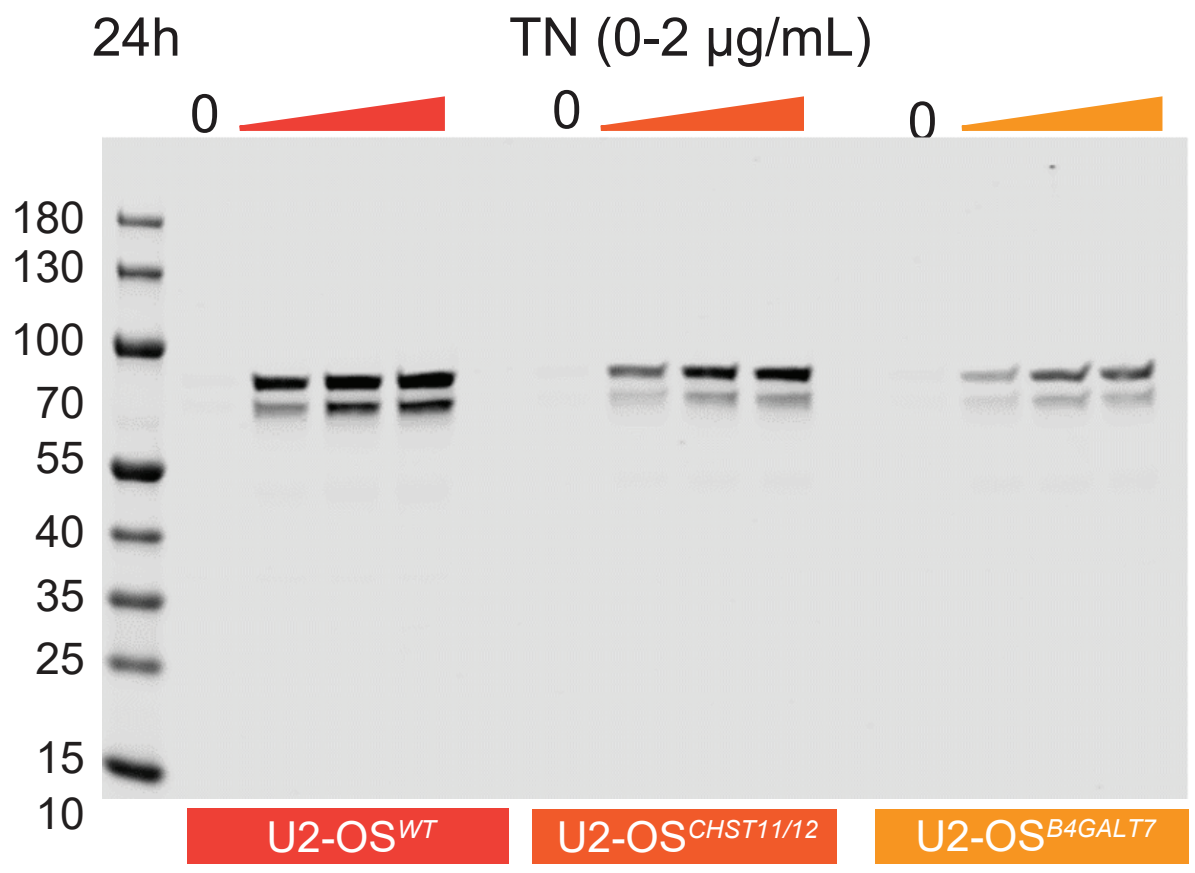


anti-GRP78

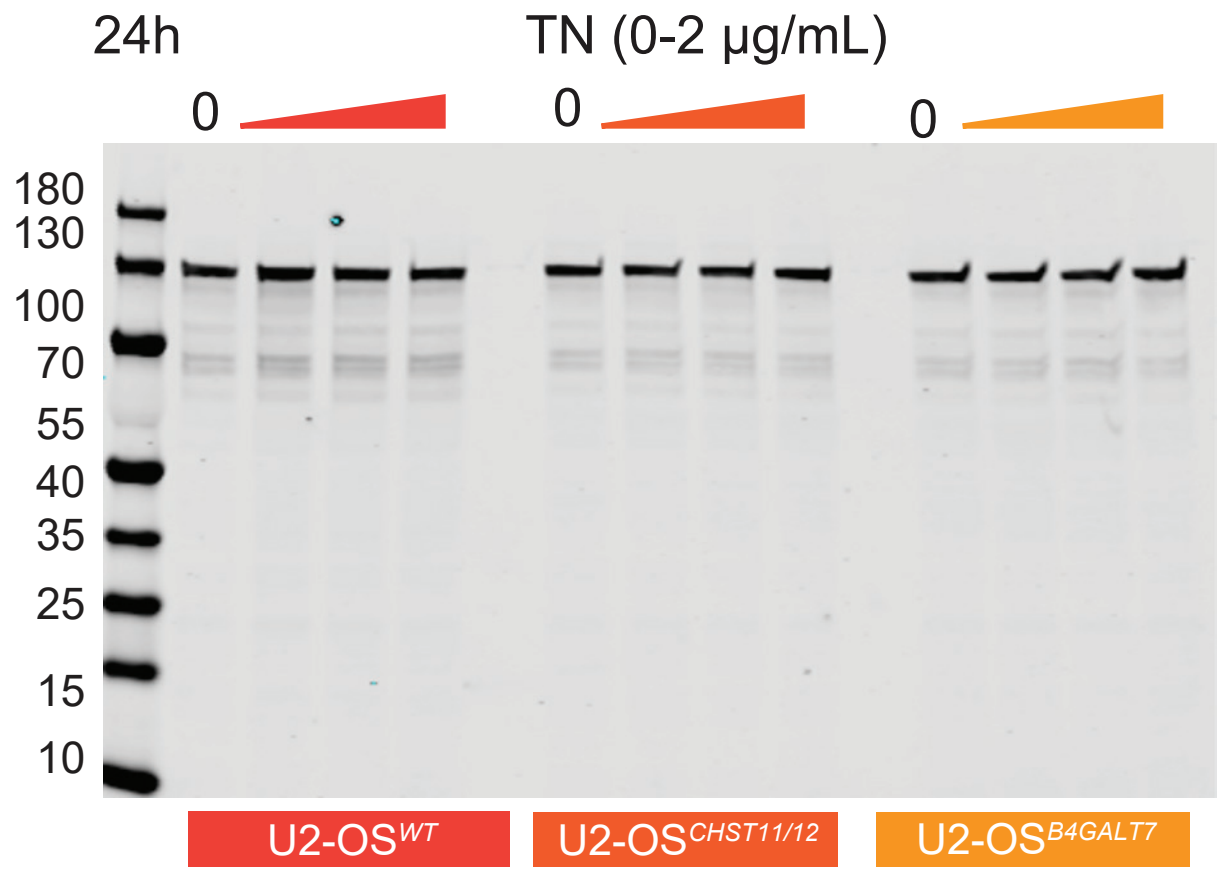


anti-vinculin

# Figure 5 uncropped western blots

For 5C

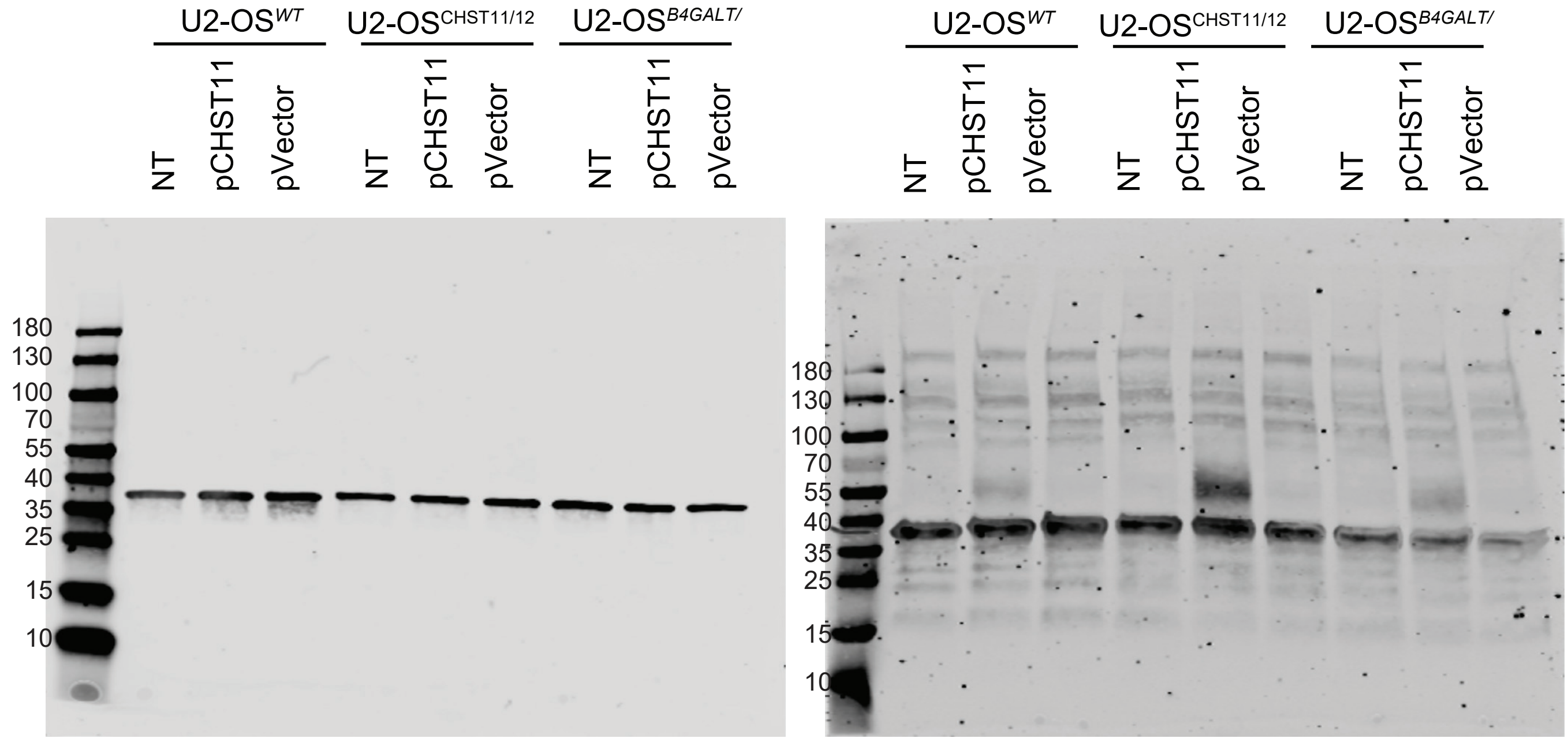


anti-GAPDH
(10 ug/well)

anti-CHST11
(50 ug/well)
