# Supplementary material for: Chondroitin sulfate shapes the unfolded protein response in osteosarcoma to sustain cancer cell survival during ER stress
Source: Cell Death Dis. 2026 Aug 1;17(1):840. doi: 10.1038/s41419-026-09151-9 (PMC13620160; doi:10.1038/s41419-026-09151-9)
Supplement: Supplementary file 2 [file 41419_2026_9151_MOESM18_ESM.docx]

# KEY RESOURCES TABLE

The table highlights the reagents, genetically modified organisms and strains, cell lines, software, instrumentation, and source data **essential** to reproduce results presented in the manuscript. Depending on the nature of the study, this may include standard laboratory materials (i.e., food chow for metabolism studies, support material for catalysis studies), but the table is **not** meant to be a comprehensive list of all materials and resources used (e.g., essential chemicals such as standard solvents, SDS, sucrose, or standard culture media do not need to be listed in the table). **However, please note that items in the table must also be reported in the method details section within the context of their use.**

**ALL references cited in the key resources table must be included in the main references list.** Citations should be formatted as “Author name et al.^#^” (e.g., Smith et al.^1^), with the citation number matching that in the main references list.

Please report the information as follows:

- **REAGENT or RESOURCE:** Provide the full descriptive name of the item so that it can be identified and linked with its description in the manuscript (e.g., provide version number for software, host source for antibody, strain name). See the sample tables at the end of this document for examples of how to report reagents.
  - In the **experimental models sections** (applicable only to experimental life science studies), please include all models used in the paper and describe each line/strain as model organism: name used for strain/line in paper: genotype (e.g., Mouse: OXTR^fl/fl^: B6.129(SJL)-Oxtr^tm1.1Wsy/J^).
  - The **Biological samples section** (applicable only to experimental life science studies) should list all samples obtained in this study or from commercial sources or biological repositories.
  - **You may list a maximum of 10 oligonucleotides or RNA sequences** in the table. If there are more than 10 to report, please provide this information as a supplemental document and reference the file (e.g., See Table S1 for XX) in the key resources table.
  - **Deposited data** should include both newly deposited data from this manuscript and existing datasets that were used in the manuscript.
  - Please include software and code mentioned in the method details or data and code availability section under **software and algorithms**.
  - Any item that does not fit the existing subheadings should be added to the “other” section. **Please do not add your own subheadings.**
- **SOURCE:** Report the company, manufacturer, or individual that provided the item or where the item can be obtained (e.g., stock center or repository).
  - For materials distributed by Addgene, please cite the article describing the plasmid and include “Addgene” as part of the identifier.
  - If an item is from another lab, please include the name of the principal investigator and a citation if it has been previously published.
  - If the material is being reported for the first time in the current paper, please indicate as “this paper.”
  - For software, please provide the company name if it is commercially available or cite the paper in which it has been initially described.
- **IDENTIFIER:** Include catalog numbers (entered in the column as “Cat#” followed by the number, e.g., Cat#3879S). Where available, please include unique entities such as [RRIDs](https://www.force11.org/group/resource-identification-initiative), Model Organism Database numbers, and accession numbers preceded by database abbreviations such as PDB or CCDC). Please ensure the accuracy of the identifiers, as they are essential for generation of hyperlinks to external sources when available. For more information about data sharing policies and a list of recommended data repositories for abbreviations, please see the Cell Press [Author’s guide to data sharing](https://www.cell.com/pb-assets/journals/research/cellpress/data/RecommendRepositories.pdf).
  - For antibodies, if applicable and available, please also include the lot number or clone identity.
  - For software or data resources, please include the URL where the resource can be downloaded.
  - When listing more than one identifier for the same item, use semicolons to separate them (e.g., Cat#3879S; RRID: AB_2255011).
  - If an identifier is not available, please enter “N/A” in the column.
  - ***A NOTE ABOUT RRIDs:*** we highly recommend using RRIDs as the identifier (in particular for antibodies and organisms but also for software tools and databases). For more details on how to obtain or generate an RRID for existing or newly generated resources, please [visit the RII](https://www.force11.org/group/resource-identification-initiative) or [search for RRIDs](https://scicrunch.org/resources).

Please use the empty table that follows to organize the information under the provided subheadings and skip sections that are not relevant to your study. To add a row, place the cursor at the end of the row above where you would like to add the row, just outside the right border of the table. Then press the ENTER key to add the row. Alternatively, you can right-click on your mouse and choose Insert > Insert rows above or Insert rows below. Please delete empty rows. Each entry must be on a separate row; do not list multiple items in a single table cell. Please see the sample tables at the end of this document for relevant examples in the life and physical sciences of how reagents and instrumentation should be cited.

## TABLE FOR AUTHOR TO COMPLETE

***Please do not add custom subheadings.*** *If you wish to make an entry that does not fall into one of the subheadings below, please contact your handling editor or add it under the “other*” subheading*.* ***Any subheadings not relevant to your study can be skipped.*** *(****NOTE:*** *references should be in numbered style, e.g., Smith et al.^1^)*

**Key resources table**

| REAGENT or RESOURCE | SOURCE | IDENTIFIER |
| --- | --- | --- |
| Antibodies | | |
| Anti-Chondroitin Sulfate (CS-56) | Abcam | Cat#ab11570 |
| Anti-GRP78/BiP mAb | Cell Signaling Technology | Cat#3177S; clone C50B12 |
| DISCOVERY DAB Map Kit (RUO) | Roche | Cat#760-124 |
| DISCOVERY UltraMap anti-Ms HRP detection kit | Roche | Cat#760-4313 |
| DISCOVERY CC1 | Roche | Cat#06414575001 |
| DISCOVERY Antibody diluent | Roche | Cat#05266319001 (part#760-108) |
| Ab Chondroitin sulfate (CS-56) | AMSBIO | Cat#270695 |
| Ab Human D-Heparan Sulfate | AMSBIO | Cat#370260-S; clone F69-3G10 |
| Ab Heparan Sulfate | AMSBIO | Cat#370255-1; clone F58-10E4 |
| Anti-C4S “stubs” | Cosmo Bio | Cat#CAC-PRPG-BC-M02; clone 2B6 |
| V450 Anti-Active Caspase-3 | BD Biosciences | Cat#560627; RRID: AB_1727415 |
| Goat anti-Mouse IgG (H+L) Superclonal™ Secondary Antibody, Alexa Fluor™488 | Invitrogen | Cat#A28175; RRID: AB_2536161 |
| Goat anti-Mouse IgG (H+L) Cross-Adsorbed Secondary Antibody, Alexa Fluor™ 488 | Invitrogen | Cat#A-11001; RRID: AB_2534069 |
| Goat anti-Mouse IgG (H+L) Highly Cross-Adsorbed Secondary Antibody, Alexa Fluor™ 568 | Invitrogen | Cat# A-11031; RRID: AB_144696 |
| Anti-58K Golgi protein antibody | Abcam | Cat#ab27043; clone 58K-9 |
| Anti-Vinculin antibody | Abcam | Cat#ab130007; clone VIN-54 |
| Anti-GAPDH | Cell Signaling Technology | Cat#5174S; clone D16H11 |
| Ab ATF4 | Cell Signaling Technology | Cat#11815S; clone D4B8 |
| Ab XBP-11s | Cell Signaling Technology | Cat#12782S; clone D2C1F |
| Ab IRE-alpha | Cell Signaling Technology | Cat#3294S; clone 14C10 |
| Ab PERK (total) | Cell Signaling Technology | Cat#5683S; clone D11A8 |
| Ab eiF2-alpha (total) | Cell Signaling Technology | Cat#9722S |
| Ab phospho-eiF2-alpha (Ser51) | Cell Signaling Technology | Cat#9721S |
| Anti-CHST11 antibody | Sigma-Aldrich | Cat#WH0050515M1; clone 4F1 |
| Anti-V5 Tag Antibody | Invitrogen | Cat#R960-25 (previously sold as cat#46-0705, cat#R960CUS, cat#46-1157); clone SV5-Pk1; RRID: AB_2556564 |
| Anti V5-Tag | Cell Signaling Technology | Cat#80076; clone: E9H80 |
| Donkey anti-Rabbit IgG (H+L) ReadyProbes™ Secondary Antibody, Alexa Fluor™ 488 | Invitrogen | Cat#R37118; RRID: AB_2556546 |
| V5 Tag Monoclonal Antibody, Alexa Fluor^TM^ 647 | Invitrogen | Cat#451098; RRID: AB_2532221 |
| Anti-V5 Tag antibody, FITC | Invitrogen | Cat#R963-25; RRID: AB_2556567 |
| V5 Tag Monoclonal Antibody | Invitrogen | Cat#46-070 (previously sold as cat#46-0705); clone SV5-Pk1; AB_2556564 |
| Anti-rabbit IgG, HRP-linked Antibody | Cell Signaling Technology | Cat#7074S |
| Goat anti-rabbit IgG (H+L), Alexa Fluor^TM^ 647 | Invitrogen | Cat#A-21245, RRID: AB_2535813 |
| Anti-mouse IgG, HRP-linked Antibody | Cell Signaling Technology | Cat#7076S |
| Anti-His Antibody, HRP (C.-term.) | Miltenyi Biotec | Cat#130-092-783; clone GG11-8F3.5.1 |
| IRDye^®^ 680RD Donkey anti-Mouse IgG (H+L) Secondary Antibody | LI-COR | Cat#926-68072; RRID: AB_10953628 |
| IRDye^®^ 800CW Donkey anti-Rabbit IgG (H+L) Secondary Antibody | LI-COR | Cat#926-32213; RRID: AB_621848 |
| IRDye^®^ 800CW Donkey anti-Mouse IgG (H+L) Secondary Antibody | LI-COR | Cat#926-32212; RRID: AB_621847 |
| IRDye^®^ 680RD Donkey anti-Rabbit IgG (H+L) Secondary Antibody | LI-COR | Cat#926-68073; RRID: AB_10954442 |
| Bacterial and virus strains | | |
| Sf9 Cells in Grace’s | Invitrogen | Cat#B82501 |
| High Five^TM^ Cells in Express Five^TM^ Medium | Invitrogen | Cat#B85502 |
| SHuffle^®^ T7 Express Competent *E. coli* | New England Biolabs | Cat#C3029J |
|  |  |  |
| Biological samples |  |  |
| Biomax OS804a Osteosarcoma Tissue Array | Biomax | <https://www.biomax.us/OS804> (no longer available) |
| Sarcoma Mixed TMA 2017 (SU2C) | Children’s Oncology Group | Children’s Oncology Group |
| Fetal Bovine Serum (FBS) | Gibco^TM^ | Cat#10270-106 |
| Bovine Serum Albumin (BSA) | Sigma Aldrich | Cat#A3059; CAS#  9048-46-8 |
| Chemicals, peptides, and recombinant proteins | | |
| Tunicamycin | Sigma-Aldrich | Cat#T7765; CAS#11089-65-9 |
| Necrostatin-1 | R&D Systems | Cat#2324; CAS#4311-88-0 |
| Dimethyl sulfoxide | Sigma-Aldrich | Cat#D2438; CAS#67-68-5 |
| Cyclohexamide | Selleck Chemicals | Cat#S7418; CAS#66-81-9 |
| Thapsigargin | Enzo Life Sciences | Cat#BML-PE180; CAS#67526-95-8 |
| Pan Caspase Inhibitor Z-VAD-FMK | R&D Systems | Cat#FMK001 |
| Ferrostatin-1 | Cayman Chemical | Cat#17729; CAS#347174-05-4 |
| Trolox | Sigma-Aldrich | Cat#238813; CAS#53188-07-1 |
| *N*-acetyl-L-cysteine (NAC) | Sigma-Aldrich | Cat#A9165; CAS#616-91-1 |
| Recombinant Subtilase AB (subAB) | Drs. A. W Paton and J. C. Paton from School of Molecular and Biomedical Science, University of Adelaide, Australia | N/A |
| Inert Subtilase AB (SubA_A272_B) | Drs. A. W Paton and J. C. Paton from School of Molecular and Biomedical Science, University of Adelaide, Australia | N/A |
| Chondroitin 4-sulfate sodium salt from bovine trachea | Sigma-Aldrich | Cat#27042; CAS#39455-18-0 |
| Chondroitinase ABC from *Proteus vulgaris* | Sigma-Aldrich | Cat#C3667; CAS#9024-13-9 |
| Chondroitinase ABC | This paper |  |
| Lipofectamine^TM^ 3000 Transfection Reagent | Invitrogen | Cat#L3000075 |
| Corning^®^ CellStripper^TM^ | Corning | Cat#25-056-CI |
| Recombinant VAR2 (DBL1-ID2a) | This paper | As in PMID: **22570492,** PMID: **26461094** |
| Recombinant DBL4 | This paper | As in PMID: **26461094** |
| Recombinant F8 scFv^2^ | This paper | As in PMID: 39215044 |
| GRP78 (Bip) (HSPA5), Recombinant Protein | MyBioSource | Cat#MBS806904; AC#P11021 |
| DNase I (from bovine pancreas) | Roche | Cat#11284932001 |
| Heparinase II | IBEX Pharmaceuticals Inc. | Cat#60-018; CAS#149371-12-0 |
| Heparinase III | IBEX Pharmaceuticals Inc. | Cat#60-020; CAS#37290-86-1 |
| Heparinase I | Iduron Ltd | Cat#HEP-ENZ I |
| Heparinase II | Iduron Ltd | Cat#HEP-ENZ II |
| Heparinase III | Iduron Ltd | Cat#HEP-ENZ III |
| AMAC (2-aminoacridone) | Sigma-Aldrich | Cat#06627; CAS#  27918-14-5 |
| Paraformaldehyde solution 4% in PBS | Santa Cruz | Cat#sc-281692; CAS#30525-89-4 |
| Thiazolyl Blue Tetrazolium Bromide (MTT) | Sigma-Aldrich | Cat#M2128; CAS#298-93-1 |
| Cellstripper | Corning | Cat#25-056-CI |
| Dulbecco′s Phosphate Buffered Saline | Gibco | Cat#A1285801 |
| Glutaraldehyde Solution | Sigma-Aldrich | Cat#354400; CAS#111-30-8 |
| ProLong™ Glass Antifade Mountant | Invitrogen | Cat#P36980 |
| Crystal violet, powder | Sigma-Aldrich | Cat#C0775; CAS# 548-62-9 |
| DAPI | Invitrogen | Cat#D3571 |
| DAPI | Invitrogen | Cat#D1306 |
| Mayer’s hematoxylin | Merck | Cat#MHS32 |
| Eosin Y | Sigma-Aldrich | Cat#HT110132 |
| HistoChoice^®^ Clearing Agent | Sigma-Aldrich | Cat#H2779 |
|  |  |  |
| Critical commercial assays | | |
| Alexa Fluor^TM^ 647 NHS Ester | Invitrogen | Cat#A20006 |
| Annexin V-FITC Early Apoptosis Detection Kit | Cell Signaling Technology | Cat#6592S |
| Pierce™ BCA Protein Assay Kit | Thermo Scientific | Cat#A65453 |
| Monarch^®^ Total RNA Miniprep Kit | New England Biolabs | Cat#T2010S |
| LunaScript ® RT SuperMix Kit | New England Biolabs | Cat#E3010L |
| Luna ® Universal qPCR Master Mix | New England Biolabs | CatM3003L |
| Deposited data | | |
|  |  |  |
|  |  |  |
| Experimental models: Cell lines | | |
| MNNG (including eGFP expressing MNNGs) | ATCC | ATCC® CRL 1547 |
| SJSA-1 | Kindly provided by Dr. Tim Triche (Children’s Hospital Los Angeles, USA) |  |
| OST | Kindly provided by Dr. Tim Triche (Children’s Hospital Los Angeles, USA) |  |
| MG-63 | ATCC | ATCC® CRL-1427 |
| MG-63.3(RRID: CVCL_WL01) | Kindly provided by Dr. Rosandra Kaplan (Pediatric Oncology Branch, NIH, Bethesda, Maryland, USA) |  |
| HOS | ATCC | ATCC® CRL-1543 |
| Saos-2 | ATCC | ATCC® HTB-85 |
| U-2-OS (U2-OS) | ATCC | ATCC® HTB-96 |
| U2-OS*^WT^* | This paper | Based on ATCC® HTB-96 |
| U2-OS*^CHST11/12^* | This paper | Based on ATCC® HTB-96 |
| U2-OS*^B4GALT7^* | This paper | Based on ATCC® HTB-96 |
| HEK293T | ATCC | ATCC® CRL-11268 |
| Experimental models: Organisms/strains | | |
| NOD.Cg-*Prkdc^scid^*/J | The Jackson Laboratory | <https://www.jax.org/strain/001303> |
| Oligonucleotides | | |
| gRNA *B4GALT7*  TGACCTGCTCCCTCTCAACG | TAG Copenhagen ApS | N/A |
| Primer B4GALT7  *F* **5^’^-3^’^** GGCTGAGTGAAGTCAGTGCT | TAG Copenhagen ApS | N/A |
| Primer B4GALT7  R **3^’^-5^’^** ACTGCCCCATCCTTCCCA | TAG Copenhagen ApS | N/A |
| gRNA CHST11  GATAAAGGATCCCAAGCAAG | TAG Copenhagen ApS | N/A |
| Primer CHST11  *F* **5^’^-3^’^** CTCAGCACTTCCAGACCAACTC | TAG Copenhagen ApS | N/A |
| Primer CHST11  R **3^’^-5^’^** TAGGGTTGGAAGAGGCAGAAATC | TAG Copenhagen ApS | N/A |
| gRNA CHST12  CGTGTGCAAGTAGAAGTGCG | TAG Copenhagen ApS | N/A |
| Primer: CHST12  *F* **5^’^-3^’^** CTCTGCAGGAAGCTGAAGTGAG | TAG Copenhagen ApS | N/A |
| Primer: CHST12  R **3^’^-5^’^** GTCGTAGCCTCTCACGCTCT | TAG Copenhagen ApS | N/A |
| *qPCR primer S18*  *F* **5^’^-3^’^***:* TTG ACG GAAGGGCACCACCAG | Integrated DNA Technologies | N/A |
| *qPCR primer S18*  R **3^’^-5^’^**: GCACCACCACCCACGGAATCG | Integrated DNA Technologies | N/A |
| *qPCR primer RPL32*  *F* **5^’^-3^’^***:* CCCCTTGTGAAGCCCAAGA | Integrated DNA Technologies | N/A |
| *qPCR primer RPL32*  R **3^’^-5^’^**: GACTGGTGCCGGATGAACTT | Integrated DNA Technologies | N/A |
| *qPCR primer HSPA5*  *F* **5^’^-3^’^***:* TTTGCCAACAATACAAACCTAGGTA | Invitrogen | A15629 |
| *qPCR primer HSPA5*  R **3^’^-5^’^**: AGAATCCAATACTGCCCAAGGA | Invitrogen | A15630 |
| Recombinant DNA | | |
| gRNA expression plasmid | Addgene | Cat#68370 |
| Cas9-PBKS plasmid (GFP tagged) | Addgene | Cat#68371 |
| pCMV3-C-His Negative Control Vector (C-terminal His-tagged) | Sino Biological | Cat#CV015 |
| pCMV3-human CHST11 / C4ST-1 transcript variant 2 Gene ORF-C-His (C-terminal His-tagged) | Sino Biological | Cat#HG11396-CH |
|  |  |  |
|  |  |  |
| Software and algorithms | | |
| FlowJo v10 | Becton, Dickson & Company | [Home \| FlowJo, LLC](https://www.flowjo.com/) |
| R: The R Project for Statistical Computing | R: The R Project for Statistical Computing | [R: The R Project for Statistical Computing](https://www.r-project.org/) |
| ZEN Microscopy Software (black edition) | Zeiss | [Zeiss](https://www.zeiss.com/) |
| ZEN Microscopy software (blue edition) | Zeiss | [Zeiss](https://www.zeiss.com/) |
| Image J | Image J | [ImageJ](https://imagej.net/ij/) |
| SlidePath Digital Imaging Hub | Leica | N/A |
| InCuCyte Software (v2020C) | Sartorius | [Incucyte Software v2020c \| Sartorius](https://www.sartorius.com/en/products/live-cell-imaging-analysis/live-cell-analysis-software/incucyte-software-v2020c?utm_source=google&utm_medium=cpc&utm_campaign=incucyte&utm_term=brand&utm_content=search&gad_source=1&gad_campaignid=21102879574&gclid=CjwKCAjw7fzDBhA7EiwAOqJkhyU-ZXUjUIP_1g-J3gSWl091ni40HdEx1oekPLEKcCBiTOJosPqzihoCQNkQAvD_BwE) |
| ViiA^TM^ software | Thermo Fisher Scientific | [Applied Biosystems ViiA™ 7 Real-Time PCR System \| Thermo Fisher Scientific - DK](https://www.thermofisher.com/dk/en/home/technical-resources/software-downloads/applied-biosystems-viia-7-real-time-pcr-system.html) |
| CRISPR Design Tool | Horizon | [Horizondiscovery](https://horizondiscovery.com/en/ordering-and-calculation-tools/crispr-design-tool) |
| Other | | |
| Sepharose 6B-L column (70 cm) | Cytivia | Cat#17016001 |
| Poly-Prep® Chromatography Columns 9 cm high, 2 ml bed volume (0.8 x 4 cm) | Biorad | Cat#7311550 |
| HisTrap HP column | Cytivia | Cat#17-5248-02 |
| Ultima Gold XR | PerkinElmer Life Sciences | Cat#6013119 |
| High Precision Cover Glass 1.5H, 12mm | Azer Scientific Inc | Cat#ES0117520 |
| Pall Microsep Advance Centrifugal Devices with Omega Membrane (MWCO 30K) | Fisher Scientific | Cat#MCP030C41 |
| Absolute Ethanol (>99,85) | VWR | Cat#20821.310 |
| Ethanol (96%) | VWR | Cat#20824.365 |
| Ethanol (70%) | VWR | Cat#83801.290 |
| Triton X-100 | VWR | Cat#9002-93-1 |
