# Supplementary material for: Chondroitin sulfate shapes the unfolded protein response in osteosarcoma to sustain cancer cell survival during ER stress
Source: Cell Death Dis. 2026 Aug 1;17(1):840. doi: 10.1038/s41419-026-09151-9 (PMC13620160; doi:10.1038/s41419-026-09151-9)
Supplement: Supplementary file 3 [file 41419_2026_9151_MOESM19_ESM.docx]

**SUPPLEMENTARY FIGURE LEGENDS**

**S1: (A)** Representative immunohistochemistry images of human osteosarcoma specimens stained with rVAR2-V5 detected by anti-V5-HRP (n=75 intact cores), showing low and high ofCS positivity. The scale bars represent 100 μm. **(B)** Human osteosarcoma cells were analyzed by flow cytometry for V5-tagged recombinant VAR2 (rVAR2-V5) or rDBL4-V5 (rCtrl) binding at the indicated protein concentrations (200, 100, 50, 25, or 12.5 nM) in the absence or presence of soluble Chondroitin sulfate A (CSA) chains (400 μg/mL) as detected by anti-V5-FITC. Graphs show geometric Median Fluorescence Intensity (MFI). Data are representative of n=3-10.

**Table S1.** Table summarizing clinical characteristics (sex and tumor stage) and IHC ofCS scores for samples in the Biomax OS804a tissue microarray (TMA).

**S2: (A)** Representative images showing the scoring used for GRP78 antibody (0-3 scoring) in the Sarcoma Mixed TMA 2017 (SU2C). Scalebar represents 50 µm. **(B)** GRP78 antibody optimization on formalin fixed paraffin embedded MNNG cells 48 h post 6 h treatment with thapsigargin (TG, 50 nM) or vehicle control (DMSO). Scalebar represents 50 µm. **(C)** CS-56 antibody optimization on formalin fixed paraffin embedded U2-OS^WT^ and U2-OS*^CHST11/12^* cells. Scalebar represents 50 µm. **(D)** Representative images showing the scoring used for CS-56 antibody (0-3 scoring) in the Sarcoma Mixed TMA 2017 (SU2C). Scalebar represents 50 µm.

**Table S2.** Clinical characteristics of osteosarcoma patients represented in the Sarcoma Mixed TMA 2017 (SU2C) (n=22), used for CS-56 and GRP78 immunohistochemical staining.

**S3:** (**A**) Heatmap showing normalized mRNA expression of canonical Unfolded Protein Response signaling components in osteosarcoma tumors from GSE19276 used to categorize the tumors into ER stress high and ER stress low profiles. **(B)** mRNA expression levels of selected key enzymes involved in chondroitin sulfate synthesis in ER stress high or ER stress low profiled osteosarcoma tumors (from GSE19276).

**S4:** Heatmap showing normalized mRNA expression of known chondroitin sulfate proteoglycans, heparan sulfate chondroitin sulfate synthesizing enzymes, proteoglycans, and canonical Unfolded Protein Response signaling components in osteosarcoma tumors (from GSE19276).

**S5: (A)** Western blot for GRP78 expression in MNNG cells recovering after 6 h thapsigargin (TG, 50 nM or 100 nM) or vehicle (DMSO) 6 h (pulse) for up to 120 h (as indicated on the infographics above the blots). Vinculin was used as protein loading control. **(B)** Western blot for GRP78 expression in MNNG cells exposed to TG (50 nM) or vehicle (DMSO) for 6 h following by 24 h or 48 h recovery. Vinculin was used as protein loading control. Data are representative of n=3. **(C)** Cell surface CS detection by CS-56 binding to MNNG cells recovering (48 h) from TG/DMSO pulse as detected by flow cytometry, graph represents mean MFI ± SEM, n=3. **(D)** Confocal microscopic images of MNNG cells treated for 24 h with tunicamycin (50 ng/mL) or vehicle (DMSO) and stained with anti-58K golgi (red) or rVAR2-V5 (green) detected by Alexa 594 or Alexa 488, respectively, after permeabilization. Nuclei were visualized with DAPI. Scalebar indicates 20 µm. **(E)** Confocal microscopic images of MNNG cells exposed to heat shock (46 ^o^C) for 35 min and stained with anti-58K golgi (red) or rVAR2-V5 (green) after permeabilization. Nuclei are stained with DAPI.

**S6: (A)** Table Sanger sequencing electropherogram showing DNA sequence analysis of U2-OS knockout cells. **(B)** Microgram 4-*O* sulfated chondroitin sulfate relative to total CS in U2-OS parental (U2-OS*^WT^)* and knockout cell lines (U2-OS*^CHST11/12^* and U2-OS*^B4GALT7^*) as measured by HPLC. **(C)** Western blot analyses of GRP78 expression in U2-OS isogenic cell lines treated with tunicamycin (TN, 0.2, 1, 2 µg/ml) or vehicle/DMSO (0) for 6 h (upper), 8 h (middle), or 24 h (lower). Vinculin was used as protein loading control. All blots from 3 biological replicates are shown. Red markings indicate blots that were developed at the same time for relative band intensity reference in replicate 2.

**S7:** Western blot analyses of GRP78 expression in U2-OS isogenic cell lines treated with thapsigargin (TG, 50, 100, 200 nM) or vehicle/DMSO (0) for 6 h (upper), 8 h (middle), or 24 h (lower). Vinculin was used as protein loading control. All blots from 3 biological replicates are shown (A, B and C). Red markings indicate blots that were developed at the same time for relative band intensity reference in replicate 2.

**S8: (A)** Western blot analyses of soluble GRP78 (sGRP78:media) or cellular (GRP78:lysate) in U2-OS isogenic cell lines treated with thapsigargin (TG, 100 nM) or vehicle (DMSO). Recombinant GRP78 (rGRP78) was used as positive control for media supernatant. Vinculin was used as protein loading control for lysate. Blots are representative of n=3. **(B)** Densitometric quantification of sGRP78 protein levels over time, normalized to total protein measured by BCA. Data shown are mean ± SEM of n=3 (non-significant, 2-way ANOVA).

**S9:** qPCR analysis of *HSPA5* (GRP78 gene) mRNA levels in U2-OS isogenic cell lines treated with **(A)** tunicamycin (TN (0.2 µg/mL or 1 µg/mL)) or **(B)** thapsigargin (TG (50 nM or 100 nM)) for 6 h or 24 h. Graphs represent mean fold change of normalized RQ to two house holding genes (RPL2 and S18), n=3 ± SEM. (WT versus CS KO cells are non-significant in 2-Way ANOVA).

**S10: (A)** Densitometric quantification of GRP78 protein levels in U2-OS isogenic cells exposed to vehicle (DMSO) and CHX over time, normalized to total protein and represented relative to time 0. Data shown are mean ± SD of n=3 (non-significant, 1-way ANOVA). **(B)** Representative (upper) Ponceau (total protein) and (lower) Western blot analysis of GRP78 expression in U2-OS isogenic cells. **(C)** Densitometric quantification of GRP78 protein levels in U2-OS isogenic cells exposed to thapsigargin (TG, 100 nM) and CHX over time, normalized to total protein and represented relative to time 0. Data shown are mean ± SD of n=3 (non-significant, 1-way ANOVA). **(D)** Representative (upper) Ponceau (total protein) and (lower) Western blot analysis of GRP78 expression in U2-OS isogenic cells.

**S11:** Real-time death assessment (PI uptake/red area) in the U2-OS isogenic cells treated with **(A)** thapsigargin (TG, 500 nM) or **(B)** tunicamycin (TN, 2 ug/mL) using IncuCyte live imaging system. Data represents the mean of 3 technical replicates, representative of n=3-6.

**S12**: (**A**) Western blot analysis of CHST11 expression in MNNG and MG-63 cells transfected (48h h) with CHST11 plasmid (pCHST11) or empty control plasmid (pVector). GAPDH was used as protein loading control. Normalized death (relative to time 0) measured real-time in MNNG cells transfected with pVector or pCHST11 and treated with (**B**) TG (50 nM) or (**C**) TN (100 ng/mL) or vehicle (DSMO) using IncuCyte. (**D**) Death levels in MNNG cells at 72 h post TG/TN treatment. P-values obtained by unpaired T-test. Normalized death (relative to time 0) measured real-time in MG-63 cells transfected with pVector or pCHST11 and treated with (**E**) TG (1 µM) or (**F**) TN (4 µg/mL) or vehicle (DSMO) using IncuCyte. (**G**) Death levels in MG-63 cells at 72 h post TG/TN treatment. P-values obtained by unpaired T-test.

**S13: (A)** Cell death quantifications at 32 h when treated with Q-VD-OPh (20 µM) ± TN (1 ug/mL TN) or vehicle using the IncuCyte system. Graphs show mean red area ± SEM of 3 technical replicates, representative of n = 3. **(B)** Cell death quantifications at 32 h when treated with Z-VAD-FMK (10 µM) ± TN (1 ug/mL). Graphs show mean red area ± SEM of 3 technical replicates, representative of n = 3.

**S14:** Real-time death assessment in the U2-OS isogenic cell lines (U2-OS^WT^ (left), U2-OS*^CHST11/12^* (middle), and U2-OS*^B4GALT7^* (right) cells) using the IncuCyte system. **(A)** Cell death quantifications at 32 h when treated with NST-1 (30 µM), tunicamycin (TN) (1 µg/mL), or both in combination. Graphs show mean of 3 technical replicates ± SEM, representative of n=3, P=0.0208, 1 way ANOVA. **(B)** Cell death quantifications at 32 h when treated with NST-1 (30 µM), thapsigargin (TG) (200 nM), or both in combination. Graphs show mean of 3 technical replicates ± SEM, representative of n=3; **(C)** Cells were treated with FST-1 (4 µM), TN (1 µg/mL), or both in in combination. Graphs show mean of 3 technical replicates ± SEM, representative of n=3. P=0.0141 1-Way ANOVA. **(D)** Cells were treated with FST-1 (4 µM), TG (200 nM), or both in combination. Graphs show mean of 3 technical replicates ± SEM, representative of n=3.

**S15: (A)** Graph showing live colony formation in soft agar of U2-OS isogenic cell lines treated with antioxidants N-acetyl cysteine (NAC, 5 mM) or Trolox (25 µM). **(B)** Representative images of colony formation in soft agar for A. Scalebar represents 500 µm. **(C)** Hematoxylin and Eosin (H&E) or immunofluorescence images of FFPE MG63.3-*e*GFP PuMA lung sections stained with anti-GRP78/BiP monoclonal antibody (pink) or anti-ofCS scFv2 (F8, red) with or without chondroitinase ABC (chABC) treatment; cell nuclei were counterstained with DAPI (blue). **(D)** Quantifications of MNNG-*e*GFP micro metastasis burden in the *ex vivo* pulmonary metastasis assay (PuMA) relative to day 0 (biological experiment displayed in figure 5). Cells were pre-coated with rVAR2 (400 nM) or chondroitinase ABC (chABC, 0.2 mg/mL) digested prior to inoculation. **(E)** DAPI uptake assessed using flow cytometry upon rVAR2 (400 nM) or CHase treatment (0.2 mg/mL) of MNNG cells. Cell fractions are displayed as DAPI negative (live) or DAPI positive (dead). **(F)** MNNG cells continuously cultured in the presence of rVAR2 for 72h. Cell survival assessed by crystal violet assay.

**S16:** **(A)** Representative images of MNNG-*e*GFP micro metastatic burden in the *ex vivo* pulmonary metastasis assay (PuMA) 2^nd^ repeat. Cells were pre-coated with rVAR2 or chondroitinase ABC (chABC) digested prior to inoculation. Images were taken at 20X magnification. **(B)** MNNG-*e*GFP pulmonary metastatic burden over time relative to day 0, 10 lung pieces/group. **(C)** Quantifications of MNNG-*e*GFP pulmonary metastatic burden at day 0 and **(D)** day 14. P=0.0470 and P=0.0140 (1-way ANOVA).

**S17:** **(A)** Representative images of MNNG-*e*GFP micro metastatic burden in the *ex vivo* pulmonary metastasis assay (PuMA) 3^rd^ repeat. Cells were pre-coated with rVAR2 prior to inoculation. Images were taken at 20X magnification. **(B)** MNNG-*e*GFP pulmonary metastatic burden over time relative to day 0, 6-7 lung pieces/group. **(C)** Quantifications of MNNG-*e*GFP pulmonary metastatic burden at day 0 and **(D)** day 14. P=0327 (Students t-test).
