# Supplementary material for: Chondroitin sulfate shapes the unfolded protein response in osteosarcoma to sustain cancer cell survival during ER stress
Source: Cell Death Dis. 2026 Aug 1;17(1):840. doi: 10.1038/s41419-026-09151-9 (PMC13620160; doi:10.1038/s41419-026-09151-9)
Supplement: Supplementary file 4 [file 41419_2026_9151_MOESM20_ESM.docx]

**Table S1. Osteosarcoma Cohort Characteristics**

Osteosarcoma (n=75)

|  | **Sex** | | **Stage** | |
| --- | --- | --- | --- | --- |
|  | M | F | I | II |
| CS^Low^ (15 %) | 6 | 5 | 11 | 0 |
| CS^High^ (85 %) | 45 | 19 | 7 | 57 |
