# Supplementary material for: Chondroitin sulfate shapes the unfolded protein response in osteosarcoma to sustain cancer cell survival during ER stress
Source: Cell Death Dis. 2026 Aug 1;17(1):840. doi: 10.1038/s41419-026-09151-9 (PMC13620160; doi:10.1038/s41419-026-09151-9)
Supplement: Supplementary file 5 [file 41419_2026_9151_MOESM21_ESM.docx]

**Table S2 Osteosarcoma patient information**

| **Characteristics** | **Classification** | **Count** | **%** |
| --- | --- | --- | --- |
| Age at enrollment (days) (mean (SD)) |  | 5467.77 (1265.3) |  |
| Sex | Male | 13 | 59.1 |
|  | Female | 9 | 40.9 |
| Race | Asian | 0 | 0 |
|  | Black or African American | 2 | 9.1 |
|  | Native Hawaiian or Other Pacific Islander | 2 | 9.1 |
|  | Unknown or not reported | 2 | 9.1 |
|  | White | 16 | 72.7 |
| Ethnicity | Hispanic or Latino | 3 | 13.6 |
|  | Not Hispanic or Latino | 19 | 86.4 |
|  | Unknown (Individuals not reporting ethnicity) | 0 | 0 |
| Extent of disease at diagnosis (%) | Metastatic | 4 | 18.2 |
|  | Non metastatic | 18 | 81.8 |
